# Promoter methylation of DNA damage repair (DDR) genes in human tumor entities: *RBBP8*/*CtIP* is almost exclusively methylated in bladder cancer

**DOI:** 10.1186/s13148-018-0447-6

**Published:** 2018-02-06

**Authors:** Jolein Mijnes, Jürgen Veeck, Nadine T. Gaisa, Eduard Burghardt, Tim C. de Ruijter, Sonja Gostek, Edgar Dahl, David Pfister, Sebastian C. Schmid, Ruth Knüchel, Michael Rose

**Affiliations:** 1 Institute of Pathology, RWTH Aachen University, Pauwelsstr. 30, 52074 Aachen, Germany; 2 Division of Medical Oncology, Maastricht University Medical Centre, Maastricht, The Netherlands; 3 GROW—School for Oncology and Developmental Biology, Maastricht University Medical Centre, Maastricht, The Netherlands; 4 RWTH Centralized Biomaterial Bank (RWTH cBMB), Medical Faculty, RWTH Aachen University, Aachen, Germany; 5 Department of Urology, RWTH Aachen University, Aachen, Germany; 6 Department of Urology, Uro-Oncology, Robot Assisted and Reconstructive Urologic Surgery, University Hospital Cologne, Cologne, Germany; 7 Department of Urology, Klinikum rechts der Isar, Technical University Munich, Munich, Germany

**Keywords:** RBBP8/CtIP, DNA repair, Bladder cancer/BLCA, Epigenetics, Infinium HumanMethylation450 BeadChip, Urine biomarker

## Abstract

**Background:**

Genome-wide studies identified pan-cancer genes and shared biological networks affected by epigenetic dysregulation among diverse tumor entities. Here, we systematically screened for hypermethylation of DNA damage repair (DDR) genes in a comprehensive candidate-approach and exemplarily identify and validate candidate DDR genes as targets of epigenetic inactivation unique to bladder cancer (BLCA), which may serve as non-invasive biomarkers.

**Methods:**

Genome-wide DNA methylation datasets (2755 CpG probes of *n* = 7819 tumor and *n* = 659 normal samples) of the TCGA network covering 32 tumor entities were analyzed in silico for 177 DDR genes. Genes of interest were defined as differentially methylated between normal and cancerous tissues proximal to transcription start sites. The lead candidate gene was validated by methylation-specific PCR (MSP) and/or bisulfite-pyrosequencing in different human cell lines (*n* = 36), in primary BLCA tissues (*n* = 43), and in voided urine samples (*n* = 74) of BLCA patients. Urines from healthy donors and patients with urological benign and malignant diseases were included as controls (*n* = 78). mRNA expression was determined using qRT-PCR in vitro before (*n* = 5) and after decitabine treatment (*n* = 2). Protein expression was assessed by immunohistochemistry (*n* = 42). R 3.2.0. was used for statistical data acquisition and SPSS 21.0 for statistical analysis.

**Results:**

Overall, 39 DDR genes were hypermethylated in human cancers. Most exclusively and frequently methylated (37%) in primary BLCA was *RBBP8*, encoding endonuclease CtIP. *RBBP8* hypermethylation predicted longer overall survival (OS) and was found in 2/4 bladder cancer cell lines but not in any of 33 cancer cell lines from entities with another origin like prostate. *RBBP8* methylation was inversely correlated with RBBP8 mRNA and nuclear protein expression while RBBP8 was re-expressed after in vitro demethylation. *RBBP8* methylation was associated with histological grade in primary BLCA and urine samples. *RBBP8* methylation was detectable in urine samples of bladder cancer patients achieving a sensitivity of 52%, at 91% specificity.

**Conclusions:**

*RBBP8* was identified as almost exclusively hypermethylated in BLCA. *RBBP8*/CtIP has a proven role in homologous recombination-mediated DNA double-strand break repair known to sensitize cancer cells for PARP1 inhibitors. Since *RBBP8* methylation was detectable in urines, it may be a complementary marker of high specificity in urine for BLCA detection.

**Electronic supplementary material:**

The online version of this article (10.1186/s13148-018-0447-6) contains supplementary material, which is available to authorized users.

## Background

Molecular characterization of cancer entities based on cohorts of tumor samples from all major organs created a wealth of data, allowing researchers to identify mutational landscapes across different cancer types. These studies provided novel insides into genomic signatures independent of tissue of origin [1], highlighting driver mutations potentially suitable for targeted therapies [2–4]. Next to druggable mutations, the cancer epigenome, known to regulate gene expression, holds clues to identify novel biomarkers and therapeutic approaches improving patient stratification. Genome-wide studies have recently identified pan-cancer DNA methylation (DNAm) patterns among diverse tumor entities, e.g., affected by genetic alterations in epigenetic regulators like *DNMT3* (reviewed in Witte et al. [5]). Still, the co-existence of unique DNAm patterns indicates that also entity-specific and subtype-specific targets of epigenetic deregulation could lead to the development of distinct methylation phenotypes contributing to tumorigenesis. These specific epigenetic aberrations, also referred to as epimutations, may uncover novel targets to improve disease management in many respects.

So far, DNA methylation is proposed as a molecular biomarker for cancer detection [6] but also as a biomarker for prediction and stratification of patients with risk of distinct clinical outcome and response to therapies [7]. Owing to this, methylation of DNA repair genes in general seems to be a good pool for prediction [8–12] of how patients respond to treatment with conventional chemotherapies as well as novel classes of targets such as poly (ADP-ribose) polymerase (PARP) inhibitors. Examples of predictive methylated genes are *MGMT* in glioma (temozolomide) [13, 14], *BRCA1* in breast cancer (PARP1 inhibitors, cisplatin, and chemotherapy) [15–18], and *PRKCDBP* in colon cancer (oxaliplatin) [19], among others.

In the presented study, we were, therefore, interested to reveal whether differential DNAm patterns of DNA repair genes of the DNA damage response (DDR) network were common epimutations across cancer entities, especially in those known to be impaired in DNA repair function, such as bladder cancer [20] which showed an essentially stagnant disease management since decades [21]. Since there is, to our knowledge, no systematic screen for hypermethylation of DNA repair genes, we performed a comprehensive candidate approach comprising 177 DDR genes [22] as targets of epigenetic deregulation in 32 tumor entities. Subsequently, we exemplarily aimed at the validation of the identified lead candidate gene, *RBBP8*, to assess potential biomarker performance based on a non-invasive detection approach.

Accumulating studies propose *RBBP8*, known to encode the endonuclease CtIP [23], as a novel susceptibility gene [24] whose functional loss increases sensitivity towards PARP1 inhibition [25, 26] similar to *BRCA1* inactivation as, for instance, recently demonstrated in a mice xenograft model of breast cancer [27]. Mechanistically, RBBP8, a nuclear located protein that is conserved among vertebrates, interacts with tumor suppressors such as BRCA1 and the pRb family members through binding sites that are frequently mutated in human cancers [24]. As part of the DDR network, RBBP8/CtIP has a proven role as key factor in regulating DNA-end resection and double-strand break (DSB) repair mechanisms [28, 29] by supporting homologous recombination (HR) [30], classical non-homologous end-joining (c-NHEJ) [31, 32], and alternative non-homologous end-joining (alt-NHEJ) [33]. Thus, RBBP8/CtIP is thought to be involved in the maintenance of genome integrity in a cell cycle- and DNA damage-dependent manner [34, 35]. Here, we demonstrate that *RBBP8* whose unique hypermethylation pattern in human bladder cancer was associated with its gene silencing might serve as a biomarker that can be accessed via urine tests.

## Results

### DNAm pattern in promoter regions of DDR genes in 32 different human cancer types

**Fig. 1:**
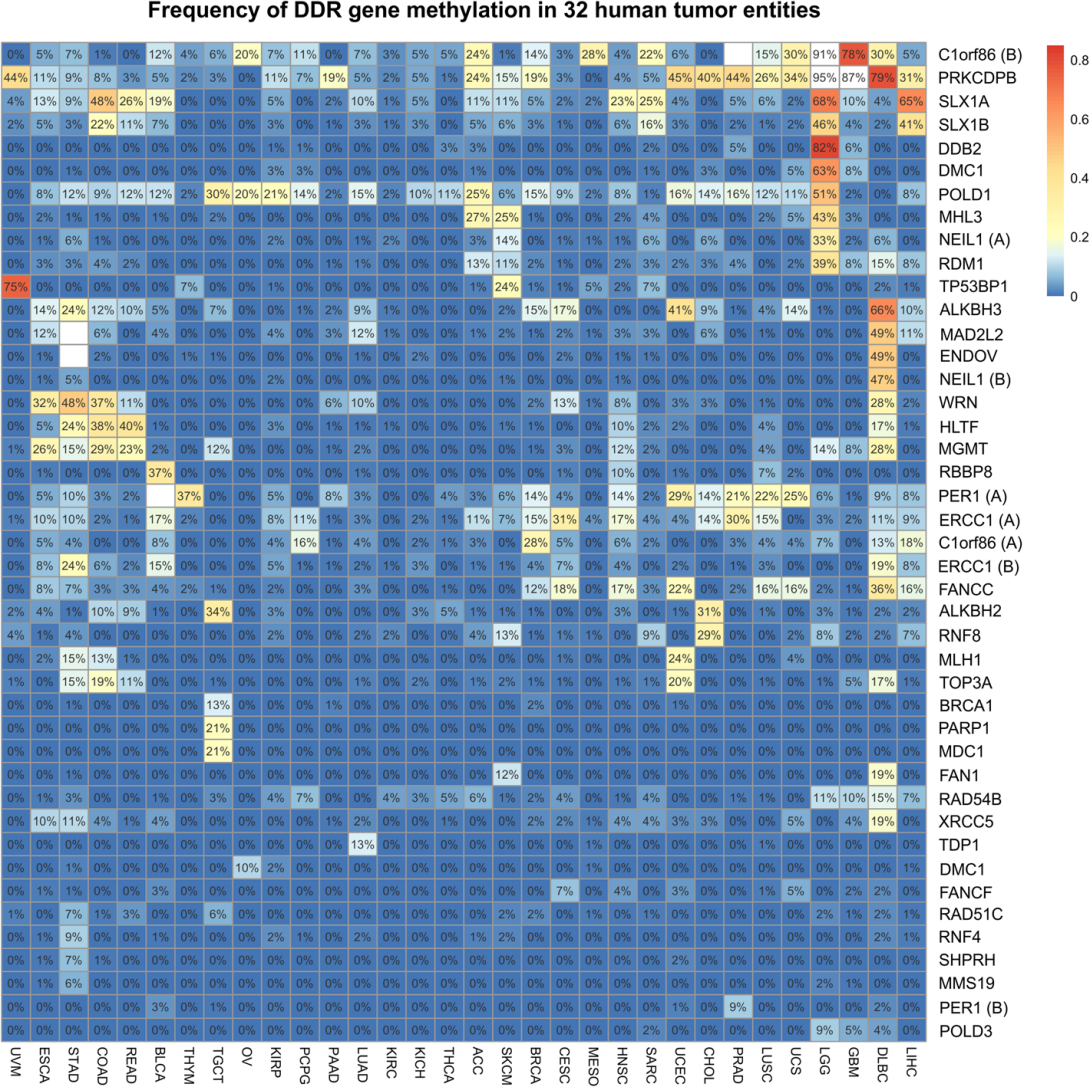
Discovery analysis of DNAm pattern in promoter regions of DNA damage repair genes across tumor entities. Promoter methylation of DDR genes was present in 32 different tumor entities. The heatmap summarizes the conducted analysis of the Infinium HumanMethylation450 BeadChip data available from the TCGA project (see the “Methods” section for details). Only those genes (CpG groups near TSS (s)) for which at least one tumor entity exhibited a hypermethylation of more than 5% of cases are shown. Fields for which corresponding normal tissues were hypermethylated in more than 15% are not shown (white). Note: The tumor entities ACC, DLBC, KICH, LGG, MESO, OV, SARC, TGCT, UCS, and UVM were included despite missing corresponding normal tissue samples. Fields were clustered hierarchically in both dimensions

Our first aim was to identify novel DNA repair genes as targets of epigenetic inactivation unique to human cancer types, which may finally be used as a non-invasive methylation biomarker. The study design is illustrated in Additional file 1. Based on the Infinium HumanMethylation450 dataset of the publically available *The Cancer Genome Atlas* (TCGA) platform, genome-wide DNA methylation data of 7819 primary tumor samples and 659 normal samples comprising 32 tumor entities (Table 1) was assessed. Overall, we performed an integrated analysis by defining CpG probe groups including initially 2755 CpG probes (sites) located between 2000 bp downstream and 500 bp upstream of the reported transcription start sites (TSS) of 177 DDR genes. Subsequently, we focused on those genes whose CpG probe set with a putative regulatory impact met the following criteria: A probe set specific and (healthy-) normalized *β* value cutoff of ~ 0.25 (*β* value ~ 0.25 (90% CI 0.20–0.35)) after transformation to *M* values (see the “Methods” section). By that, 39 DDR genes were found to show methylation in a fraction of ≥ 5% of samples in one or more entities (see heatmap in Fig. 1). This includes known epigenetic silenced DDR genes with a therapeutic impact like *BRCA1* [17, 18], *MGMT* [13, 14], and *ERCC1* [36, 37] (Additional file 2). In addition (to these already known DDR genes), we were also able to identify novel potential targets like SLX1A which has not yet been associated with DNA methylation in tumors so far. The identified DDR genes can be classified into all essential subnetworks of DNA repair mechanisms [22], i.e., mismatch repair (MMR, e.g., *MLH1*), homologous repair (HR, e.g., *RBBP8/CtiP*), dissolution of joint DNA molecules (JMs, e.g., *TOP3A*), nucleotide excision repair (NER, e.g., *ERCC1*), and base excision repair (BER, e.g., *PARP1*).

**Table 1:** TCGA studies and sample numbers used for DNA methylation analyses in this study

Study	Study name/tumor entity	Tumors	Normals
ACC	Adrenocortical carcinoma	79	0
BLCA	Bladder urothelial carcinoma	368	17
BRCA	Breast invasive carcinoma	772	96
CESC	Cervical squamous cell carcinoma and endocervical adenocarcinoma	299	3
CHOL	Cholangiocarcinoma	35	9
COAD	Colon adenocarcinoma	265	30
DLBC	Lymphoid Neoplasm Diffuse Large B-Cell Lymphoma	47	0
ESCA	Esophageal carcinoma	182	13
GBM	Glioblastoma multiforme	129	2
HNSC	Head and neck squamous cell carcinoma	481	50
KICH	Kidney chromophobe	60	0
KIRC	Kidney renal clear cell carcinoma	271	141
KIRP	Kidney renal papillary cell carcinoma	243	34
LGG	Brain lower grade glioma	498	0
LIHC	Liver hepatocellular carcinoma	360	45
LUAD	Lung adenocarcinoma	399	22
LUSC	Lung squamous cell carcinoma	337	36
MESO	Mesothelioma	82	0
OV	Ovarian adenocarcinomas	10	0
PAAD	Pancreatic adenocarcinoma	155	7
PCPG	Pheochromocytoma and paraganglioma	152	3
PRAD	Prostate adenocarcinoma	476	40
READ	Rectum adenocarcinoma	91	7
SARC	Sarcoma	244	0
SKCM	Skin cutaneous melanoma	102	1
STAD	Stomach adenocarcinoma	388	2
TGCT	Testicular germ cell tumors	149	0
THCA	Thyroid carcinoma	484	54
THYM	Thymoma	122	2
UCEC	Uterine Corpus Endometrial Carcinoma	404	45
UCS	Uterine carcinosarcoma	56	0
UVM	Uveal melanoma	80	0

We then reduced the selected list of candidates (i.e., CpG probe groups) by focusing on genes exhibiting hypermethylated in more than 15% of cases in at least one tumor entity with a minimum of ten analyzed normal tissue samples (Additional file 3). The list of genes with stringently tumor-specific hypermethylation includes, for instance, *ERCC1*, *MGMT*, *POLD1,* and *RBBP8/CtiP*. In all cases, hypermethylation was entity-specific as demonstrated by the conducted cluster analysis (see Fig. 1) and statistical testing applied to contingency tables of tumor samples (entity vs. relationship to the threshold, *p* < 10^−6^).

Finally, an association between promoter methylation of defined CpG probe sets and mRNA expression of the corresponding gene across tumor types was assessed to narrow down potential candidate genes and entities for the subsequent biomarker discovery part of this study. To ensure valid statistics, only DDR genes exceeding a methylation frequency of > 5% in identified tumor entities were included. Based on that, 9 out of the identified 14 candidate genes (see Additional file 3) passed our criteria, i.e., showed a highly significant (i.e., *p* < 0.001) association with their gene expression in one or more tumor entities like *PER1* and *POLD1* (see Additional file 4). Among others (such as *ALKBH2* and *ALKBH3*), *MGMT* promoter methylation, for instance, showed a strong inverse correlation in all analyzable tumor entities (i.e., COAD, DLBC, ESCA, GBM, HNSC, LGG, READ, STAD, TGCT).

### *RBBP8* is almost exclusively methylated in bladder cancer correlating with lower *RBBP8* mRNA expression and a favorable prognosis

**Fig. 2:**
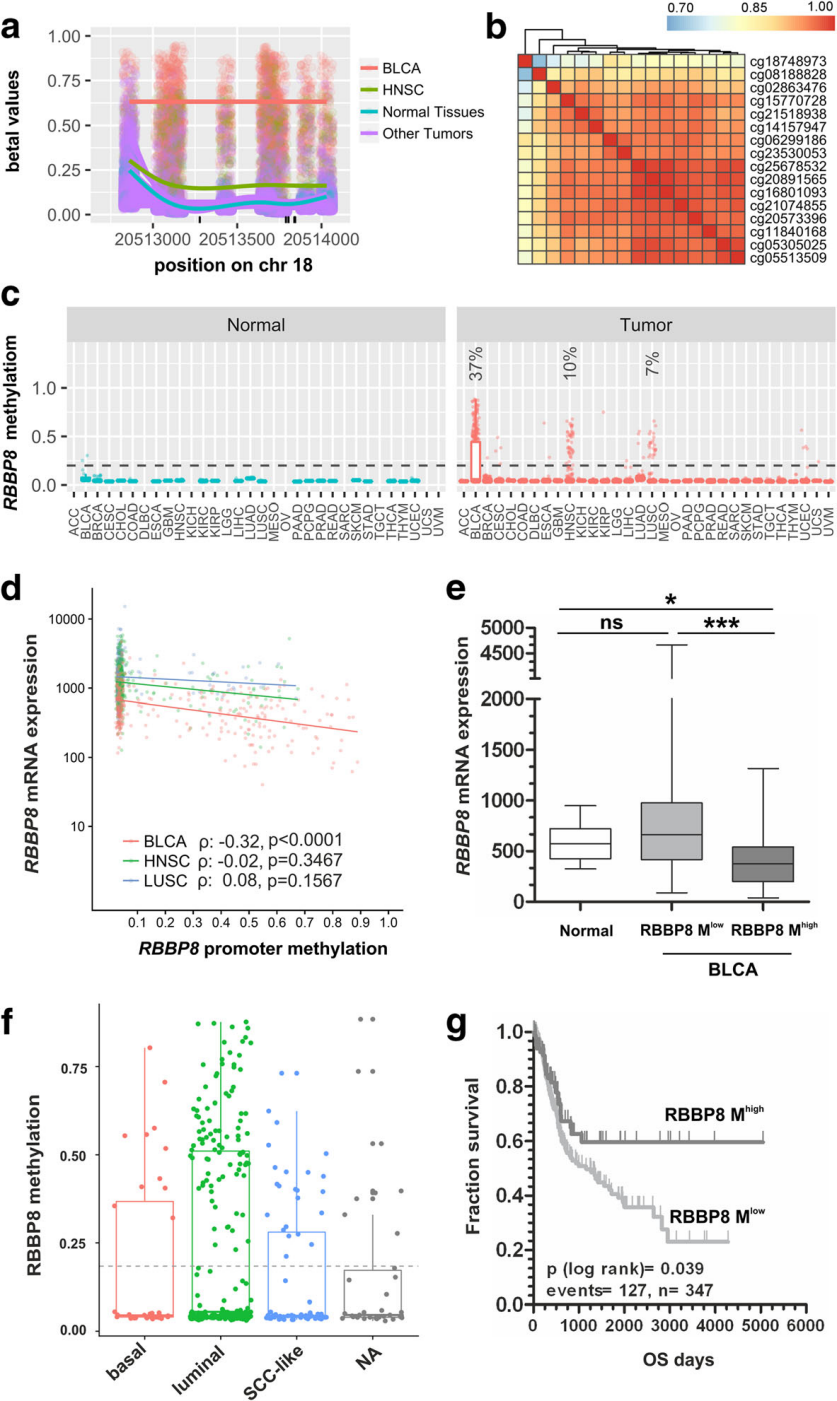
*RBBP8* promoter methylation in bladder cancer of the TCGA data set. **a** Visualization of the promoter methylation of the *RBBP8* gene as a scatterplot. The β values for each sample were jittered around the probe location and plotted as points. The per sample type 90% quantiles of methylation are shown as smoothed lines. The colors represent different sample groups (BLCA, bladder cancer; HNSC, head-neck squamous cell carcinoma). **b** The Pearson correlation coefficients (*ρ*) for probe pairs in the core region of the promoter are shown as heatmap demonstrating a high degree of correlation between probes (*ρ* > 0.7). **c** The beta values of all probes of the promoter region were summarized by their median value, stratified by the sample as well as tissue type, and visualized as a box plot. For *RBBP8* gene loci, a frequent hypermethylation (*β* > 0.25 in > 5% of cases) was only observable in 37, 10, and 7% of bladder urothelial carcinoma (BLCA), head-neck squamous cell carcinoma (HNSC), and lung squamous cell carcinoma (LUSC), respectively. **d** Inverse correlation between *RBBP8* methylation (defined CpG gene set) and *RBBP8* mRNA expression in primary bladder cancer (BLCA), neck squamous cell carcinoma (HNSC), and lung squamous carcinoma (LUSC) samples of the TCGA data portal. Spearman correlation BLCA: − 0.32, *p* < 0.001; Spearman correlation HNSC: − 0.04, *p* = ns; Spearman correlation LUSC: 0.08, *p* = ns. **e** Box plot illustrates significant downregulation of RBBP8 methylation in primary tumors featuring increased *RBBP8* promoter methylation (*β* value > 0.4). Horizontal lines — grouped medians. Boxes — 25 to 75% quartiles. Vertical lines — range, peak, and minimum. *p < 0.05, **p < 0.01, ns: not significant. **f** Box plot shows *RBBP8* methylation in primary tumors classified by intrinsic subtypes. **g** Kaplan-Meier survival curves display overall survival (OS) of patients with high *RBBP8* methylation (*β* value > 0.4, dark gray curve) compared to low *RBBP8* methylation (*β* value ≤ 0.4, gray curve) based on TCGA datasets

In bladder cancer, 3 out of these identified 39 DDR genes, i.e., *SLX1A*, *ERCC1*, and *RBBP8*, exhibited hypermethylation over 15%. Interestingly, *RBBP8* was almost exclusively methylated in bladder cancer (Fig. 2a–c). Overall, 137 out of 368 (37%) analyzed bladder tumors exhibited a tumor-specific *RBBP8* promoter methylation. The normal adjacent tissues showed methylation in 2 out of 17 cases. Nevertheless, the methylation was notably lower than that of the matching tumor tissues (*β* values 0.26 vs. 0.53 and 0.30 vs. 0.71) which might be due to an epigenetic field effect across the urothelium of bladders with cancer (e.g., [38]). Moreover, *RBBP8* promoter methylation was only present at low frequency in head and neck squamous cell carcinoma (9.6%, *n* = 46/481), lung squamous carcinoma (7.1%, *n* = 24/337), uterine carcinosarcoma (1.8%, *n* = 1/56), cervical squamous cell carcinoma (1.3%, *n* = 4/299), esophageal carcinoma (1.1%, *n* = 2/182), and lung adenocarcinoma (1.0%, *n* = 4/399). In all other analyzed cancer types, *RBBP8* methylation frequency was lower than 1.0%. A multiple *t* tests using the Holm correction for multiple comparison analysis confirmed a significantly increased methylation frequency of *RBBP8* in bladder cancer compared to all other tested tumor entities (*p* < 0.019). In addition, a significantly inverse correlation between *RBBP8* methylation (CpG probe set as defined) and *RBBP8* mRNA expression (Spearman *r* − 0.32; *p* < 0.001) was only demonstrated for BLCA (Fig. 2d). No association was observed for HNSC and LUSC, and *RBBP8* was thereof chosen as a lead candidate gene of this study for bladder cancer.

Given entity-specific methylation of *RBBP8* in BLCA, we aimed to provide a first insight on whether this epigenetic modification may provide a clinical impact in this entity. We divided the dataset (overall *n* = 405, for cohort characteristics see Additional file 5) into low methylated (RBBP8 *β* values (*β* ≤ 0.4)) and highly methylated (*β* > 0.4) tumor samples and found that a prevalent loss of *RBBP8* mRNA expression was only present in tumors with high *RBBP8* promoter methylation compared to normal bladder tissue (Fig. 2e). The close association between loss of *RBBP8* mRNA expression and *RBBP8* hypermethylation was confirmed by using a Fisher’s exact test (Table 2). Further associations of *RBBP8* methylation with clinicopathological characteristics were evaluated as well (Table 2), which showed a significant association of *RBBP8* methylation with higher histological tumor grade (*p* = 0.041). No further correlations between *RBBP8* methylation and clinicopathological characteristics were found. Classifying tumor samples by subtype, i.e., “luminal,” “basal,” and “SCC-like,” [39] *RBBP8* promoter methylation tended to be enriched in luminal-type bladder tumors (Fig. 2f).

**Table 2:** Clinicopathological parameters in relation to *RBBP8* promoter methylation of the BLCA TCGA dataset

	*RBBP8* promoter methylation^b^			
	*n* ^a^	low	high	*p* value^c^
Parameter:				
Gender				
Male	259	190	69	0.062
Female	94	78	16	
Histological tumor grade				
Low grade	20	19	1	*0.041*
High grade	330	247	83	
Tumor stage				
pT1-pT2	109	86	23	0.49
pT3-pT4	216	163	53	
pN status				
Negative	212	161	51	0.866
Positive	112	86	26	
pM status				
Negative	173	131	42	0.138
Positive	7	7	0	
*RBBP8* mRNA^d^				
Low	200	124	76	*≤ 0.001*
High	200	175	25	

Significant *p* values are in italics

^a^Only patients with primary bladder cancer without any neoadjuvant therapy were included

^b^Based on TCGA 450K DNA methylation analysis

^c^Fisher’s exact test

^d^RBBP8 mRNA expression was dichotomized at the median expression level

As dysregulation of DDR repair is known to be associated with patients’ outcome, we examined overall survival (OS) as an indicator of potential clinical impact. By Kaplan-Meier analysis, we found that patients with high *RBBP8* methylation have a longer overall survival (mean OS 3197.4 days ± 310.3; 95% CI 2589.3 to 3805.5 days) compared to low *RBBP8* methylation (mean OS 1800.6 days ± 161.4; 95% CI 1484.1 to 2117.0 days) (Fig. 2g, Table 3). The multivariate hazard ratio of 0.650 (95% CI 0.312 to 1.354, *p* = 0.249) underlines a decreased risk for tumor death, although independency statistically failed in comparison to known prognostic parameters (Additional file 6).

**Table 3:** Univariate analysis of clinicopathological parameters influencing OS

	Overall survival (OS)		
	*n* ^a^	Events	*p* value^b^
Parameter:			
RBBP8 methylation^c^			
RBBP8 M^low^	262	103	*0.039*
RBBP8 M^high^	85	24	
Gender			
Male	253	91	0.920
Female	94	36	
Histological tumor grade^d^			
Low grade	15	0	0.066
High grade	329	127	
Tumor stage			
pT1-pT2	105	23	*≤ 0.001*
pT3-pT4	214	94	
pN status			
Negative	207	53	*≤ 0.001*
Nositive	111	63	
pM status			
Negative	168	52	*0.004*
Positive	7	5	

Significant *p* values are in italics

^a^Only patients with primary bladder cancer without any neoadjuvant therapy were included

^b^Log-rank test at the two-sided significance level of 0.05

### *RBBP8* methylation is exclusively present in human bladder cancer cell lines and functionally associated with *RBBP8* mRNA expression after DAC treatment

**Fig. 3:**
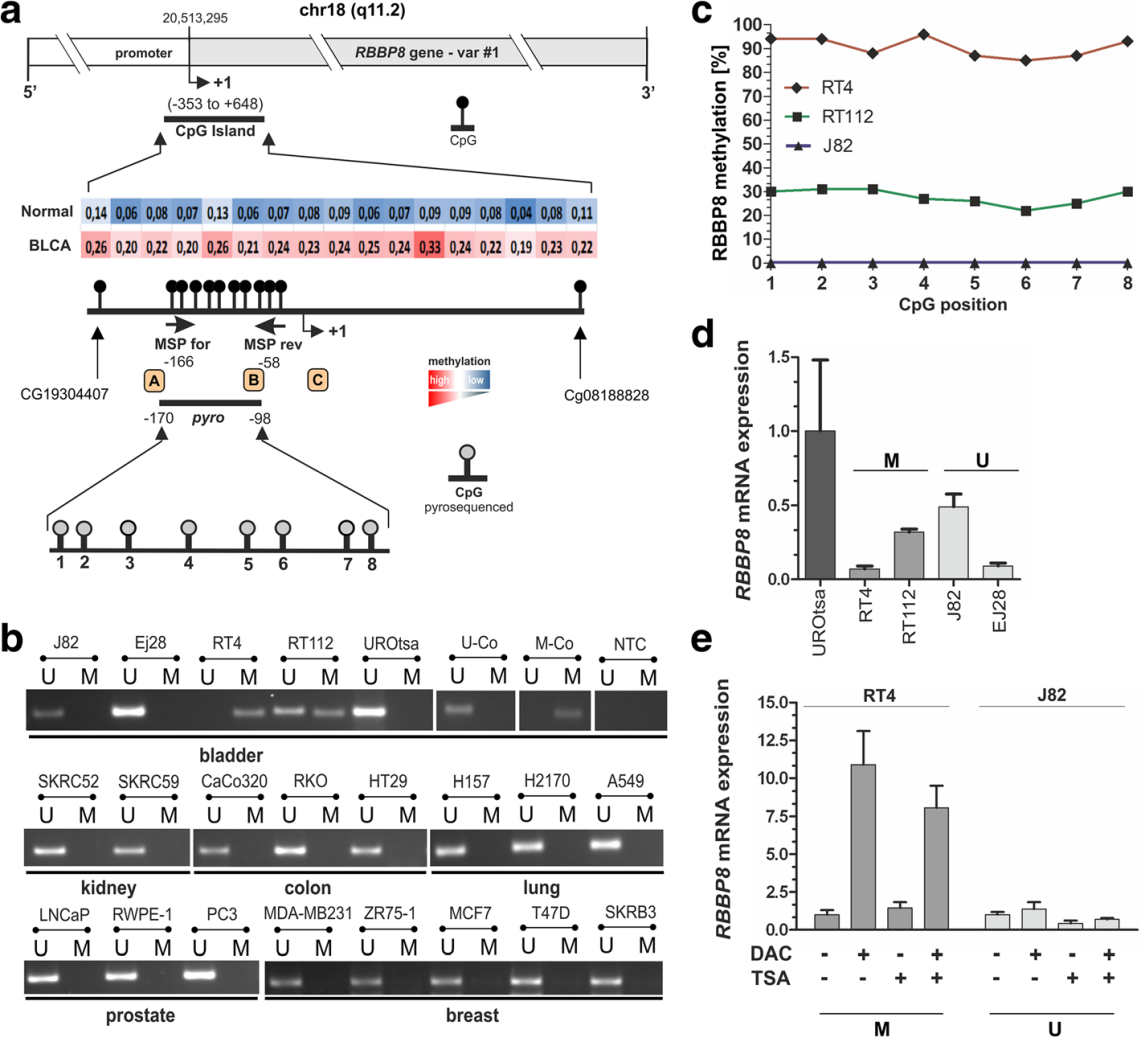
*RBBP8* promoter methylation in human cancer cell lines. **a** Schematic map of the human *RBBP8* gene including the relative positions and median *β* values of 17 CpG sites based on 450K methylation array profiling in bladder cancer (TCGA dataset) within a predicted CpG island (between base − 353 and + 648). Colored boxes present methylation level (mean *ß*-values for each CpG site) of the TCGA data set. Red, high methylation; blue, low methylation. + 1, *RBBP8* transcription start site (TSS) of variant #1. The dots indicating the methylation sites closer to where they are depicted. CpG sites analyzed by MSP (black arrows) were indicated within the upstream promoter region close to the TSS. The relative position of the promoter area analyzed by bisulfite-pyrosequencing that comprises eight single CpG sites (gray dots) is shown as a black line. Orange boxes illustrate gene transcription-relevant regulatory core and ubiquitous elements statistically identified by using Genomatix [40] software (http://www.genomatix.de/). A — core promoter motif ten elements (− 200 to − 179); B — activator protein 2 (− 109 to − 94); C — activator-, mediator-, and TBP-dependent core promoter element for RNA polymerase II transcription from TATA-less promoters (+ 28 to + 39). **b** Representative MSP results of the *RBBP8* promoter methylation status in cell lines of bladder, kidney, colon, lung, prostate, and breast cancer. Bands labeled with U and M reflect unmethylated and methylated DNA, respectively. Bisulfite-converted unmethylated, genomic (U-co), and polymethylated, genomic (M-co) DNA were used as positive controls. NTC, non-template control. **c** *RBBP8* mean methylation values of analyzed CpG sites (1 to 8) using bisulfite-pyrosequencing of bladder cancer cell lines (RT4, RT112, and J82). **d** *RBBP8* mRNA expression in normal urothelial cells (UROtsa) and bladder cancer cell lines (RT4, RT112, J82, and EJ28) arranged in relation to their *RBBP8* promoter methylation status. U, unmethylated; M, methylated. Error bars: + s.e.m. **e** qPCR analysis for *RBBP8* mRNA expression after in vitro demethylation analysis demonstrating a clear *RBBP8* re-expression after treatment with both DAC (+) and TSA (+) only in RT4 with methylated *RBBP8* promoter status (M) whereas in J82 cells without any *RBBP8* methylation (U) *RBBP8* expression was not further inducible. Non-treated cells served as controls and were set to 1. Error bars: + s.e.m.

In silico analysis of the *RBBP8* gene promoter sequence using genomic DNA information (ENSEMBL contig ENS00000101773) showed a CpG-rich island between genomic positions 20512942 and 20513943 (− 353 to + 648 bp relative to the expected transcription start site (TSS: transcript variante #1: position 20513295) on chromosome 18q which met the following criteria: DNA region ≥ 200 bp; Obs/Exp ≥ 0.6; and % GC ≥ 50. The DNA region including CpG sites that are closely associated to the TSS encodes a potential regulatory core promoter and ubiquitous elements for gene regulation as determined by in silico analysis using Genomatix [40]. In order to specify the TCGA data sets, we designed both a methylation-specific PCR (MSP) and bisulfite-pyrosequencing assay close to the TSS area to screen *RBBP8* methylation status in a large set of normal and cancer cell lines from various cancer entities. The analyzed promoter region upstream of the transcription start is illustrated in Fig. 3a.

Using MSP, a methylated *RBBP8* promoter was detected in two (RT4 and RT112) out of four bladder cancer cell lines which derived from stage 2 and higher grade bladder tumors [41], while *RBBP8* was unmethylated in normal urothelial cells (UROtsa) (Fig. 3b). The analyzed *RBBP8* promoter region was also unmethylated in 33 further cancerous cell lines, i.e., cell lines from breast cancer, lung cancer, prostate cancer, colorectal cancer, and renal cancer (see the complete list in Additional file 7). Strong *RBBP8* promoter methylation in RT4 (median methylation level 90.5%) and RT112 bladder cancer cells (median methylation level: 28.5%) was confirmed by using bisulfite-pyrosequencing covering the MSP product sequence (Fig. 3c).

In all bladder cancer cell lines (RT4, RT112, J82, and EJ28), *RBBP8* mRNA was lower expressed than in normal UROtsa cells. Lowest mRNA expression was found in RT4 cells which featured the highest methylation level as well (Fig. 3d). Functionally, we confirmed this epigenetic modification as a molecular cause for *RBBP8* gene regulation by in vitro demethylation experiments. Seventy-two hours after 5-aza-2′-deoxycytidine (decitabine (DAC)) and trichostatin A (TSA) treatment upregulation of *RBBP8* mRNA expression (FC = 11) was demonstrated in RT4 tumor cells. DAC treatment without TSA supplementation already triggered an increase in *RBBP8* mRNA expression in RT4 bladder cancer cells. Treatment with both DAC and TSA leads to a maximum of *RBBP8* mRNA re-expression. In turn, *RBBP8* mRNA was not further inducible by DAC/TSA in J82 bladder cancer cells (Fig. 3e) harboring an unmethylated *RBBP8* promoter (see Fig. 3c). These findings indicate that epigenetic alterations of the *RBBP8* gene may be caused by synergistic crosstalk between DNA methylation and histone modification.

### Validation of DNAm pattern within the *RBBP8* promoter close to the TSS in primary bladder tumors

**Fig. 4:**
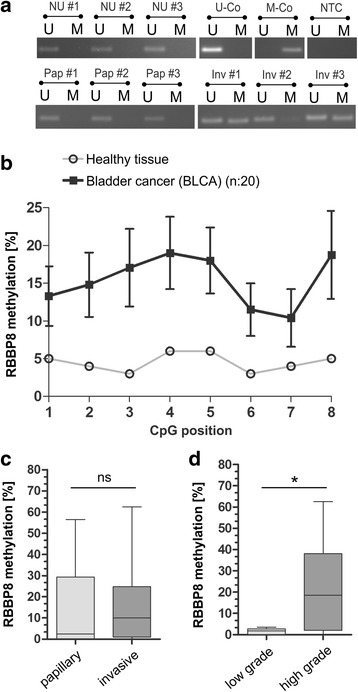
Validation of *RBBP8* promoter methylation in primary bladder tumors. **a** Representative MSP analysis shows the *RBBP8* promoter methylation status of normal urothelium (NU) and both papillary (Pap) and invasive (Inv) primary bladder cancer tissues. Band labels with U and M represent an unmethylated and methylated DNA locus, respectively bisulfite-converted unmethylated, genomic (U-co) and polymethylated, genomic (M-co) DNA were used as positive controls. NTC: non-template control. **b** *RBBP8* mean methylation values of analyzed CpG sites (1 to 8) of healthy controls and bladder tumors demonstrating tumors-specific hypermethylation. **c** to **d** Box plot analysis of *RBBP8* methylation in primary bladder tumors is based on mean values of pyrosequenced CpG sites 1–8. **c** *RBBP8* methylation shows no significant differences between the two bladder cancer pathways (papillary and invasive tumors). **d** Significant enrichment of *RBBP8* methylation is demonstrated in high-grade bladder tumors. Horizontal lines—grouped medians. Boxes—25 to 75% quartiles. Vertical lines—range, peak, and minimum; *p < 0.05. Horizontal lines—grouped medians. Boxes—25 to 75% quartiles. Vertical lines—range, peak, and minimum; ns, not significant, *p < 0.05

Based on MSP and bisulfite-pyrosequencing, *RBBP8* methylation was assessed in an independent cohort of primary bladder tumors. MSP analysis confirmed an unmethylated *RBBP8* promoter in healthy urothelial tissues. *RBBP8* methylation frequency was increased to 39.1% (9/23) in primary bladder tumors (Fig. 4a). None of the papillary low-grade tumors (*n* = 9) showed *RBBP8* methylation, whereas 9 out of 14 invasive BLCAs were methylated for the *RBBP8* locus. We subsequently analyzed the methylation status of eight CpG sites using pyrosequencing (Fig. 4b). The median *RBBP8* methylation for each CpG is shown in Fig. 4b for both normal urothelium and bladder tumors (*n* = 20). Nine out of 20 tumor (45%) samples showed a median *RBBP8* methylation level (including all eight CpGs) of > 5% (range 6 to 62.5%). Classifying the bladder tumors by papillary and invasive subtype, no clear differences in *RBBP8* promoter methylation were detected (Fig. 4c). However, a significant increased *RBBP8* promoter methylation was observed for high-grade bladder tumors, including pTa high-grade carcinomas, (mean methylation 20.9%; s.d. ± 4.4%) compared to low-grade BLCA (mean methylation 2.4%; s.d. ± 1.4%) (Fig. 4d). A close association between *RBBP8* and higher histological tumor grade (*p* = 0.041) was also confirmed by Fisher’s exact test (Additional file 8).

### RBBP8 protein expression in bladder cancer

**Fig. 5:**
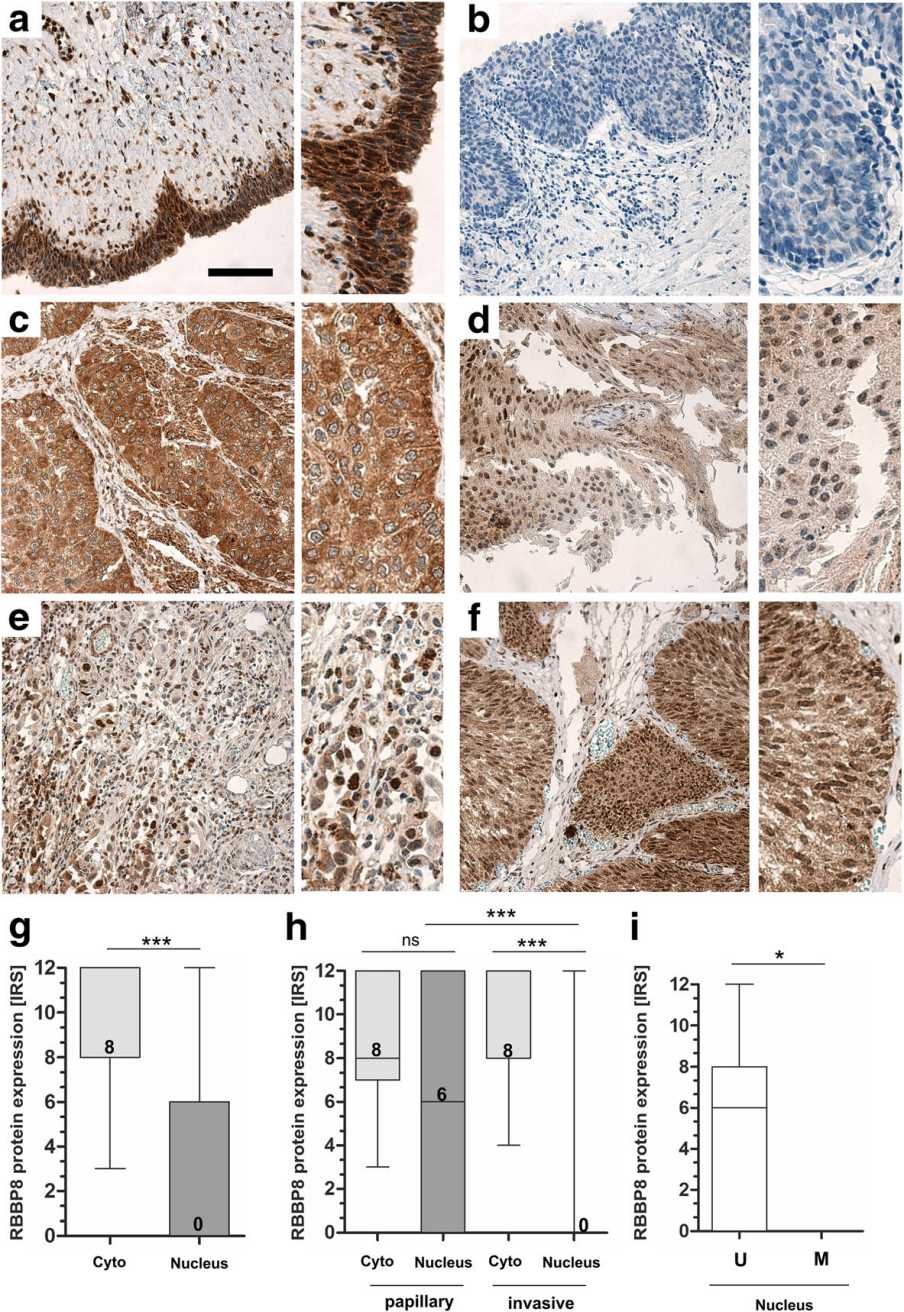
RBBP8 protein loss in nuclei of bladder tumors. Immunohistochemical RBBP8 protein staining of representative tissues are shown. **a** Strong RBBP8 immunoreactivity was detected in the cytoplasm and in the nuclei of a healthy urothelium, Scale bar: 100 μm. **b** Negative control of urothelial cell layers. The application of primary antibody was omitted. **c** Strong RBBP8 immunoreactivity in the cytoplasm of high grade, invasive tumor cells which completely lack nuclear staining. **d** Moderate cytoplasmatic and heterogeneously nuclear RBBP8 protein staining in invasive tumor cells. **e** Low RBBP8 protein expression in the cytoplasm of invasive bladder cancer showing strong RBBP8 staining in the nucleus. **f** Strong nuclear and cytoplasmic RBBP8 staining in non-invasive, papillary tumor cells. **g** Box plot demonstrating overall significant loss of RBBP8 protein only in the nucleus of bladder tumors. **h** Box plot graph illustrates the loss of RBBP8 protein within the nuclei of high-grade invasive bladder tumors. **i** Box plot shows a significant RBBP8 protein loss in tumors harboring RBBP8 promoter methylation. U, unmethylated; M, methylated. Horizontal lines — grouped medians. Boxes — 25 to 75% quartiles. Vertical lines — range, peak, and minimum; ns, not significant, *p < 0.05, ***p < 0.001

RBBP8 protein expression was characterized in normal urothelium and in bladder tumor tissues using immunohistochemistry. We found RBBP8 protein staining in the cytoplasm and frequently in the nuclei of the normal urothelium (Fig. 5a). Advanced bladder tumors showed a similar cytoplasmatic protein level, but only sporadically a nuclear protein localization was observed (Fig. 5c–e). Interestingly, low-grade non-invasive bladder tumors retained high levels of RBBP8 protein within the nucleus (Fig. 5f).

This observation was confirmed by quantification of RBBP8 protein staining according to an adapted immunoreactive score (IRS) developed by Remmele and Stegner [42] in both the cytoplasm and the nuclei. Overall, 42 bladder carcinomas were compared evaluating the average RBBP8 protein expression in a semi-quantitative manner (for cohort characteristics, see Additional file 9). Cytoplasmatic RBBP8 protein expression remained visible in all bladder tumors (median IRS, 8). In contrast, 71.4% (*n* = 30/42) of the analyzed bladder tumors exhibited a significant loss of RBBP8 protein expression in the nuclei with an IRS score below 3 (median IRS, 0) (Fig. 5g). Stratifying the tumor samples by substage, we revealed that the observed loss of nuclear RBBP8 protein significantly correlates with advanced invasive (pT1-pT4) bladder tumors, while pTa non-invasive tumors did not significantly differ in cytoplasmic and nuclear RBBP8 protein level (Fig. 5h).

We did not observe any association between cytoplasmatic RBBP8 staining and clinicopathological parameters. As RBBP8 is a known DNA repair protein that is functionally located in the nucleus (see for instance protein-staining pattern of breast cancer cells [43]), we further focused on nuclear RBBP8. Nuclear RBBP8 protein was significantly reduced in invasive tumors compared to papillary tumors (see Fig. 5h). A close association between loss of nuclear RBBP8 protein and both advanced tumor stages and high-grade BLCA was significantly illustrated by a Fisher’s exact test (Table 4). A significant downregulation of RBBP8 protein expression in nuclei was demonstrated for bladder tumors with an increased *RBBP8* promoter methylation (> 5%) compared to those with a low *RBBP8* methylation (Fig. 5i). In line, a statistically inverse correlation between nuclear RBBP8 protein level and *RBBP8* promoter methylation was confirmed (Table 4), supporting a contribution of epigenetic *RBBP8* alterations to its protein loss in primary bladder tumors.

**Table 4:** Clinicopathological parameters in relation to nuclear RBBP8 protein expression

	RBBP8 IRS^b^ nuclear				
	*n* ^a^	Low	High	*p* value^c^	Spearman *r*
Parameter:					
Age at diagnosis					
< 70 years	23	14	9	0.213	− 0.195
≥ 70 years	19	15	4		
Gender					
Male	33	25	8	0.075	− 0.278
Female	9	4	5		
Tumor subtype					
Non-invasive papillary	17	8	9	*0.012*	− 0.392
Invasive	25	21	4		
Histological tumor grade^d^					
Low grade	12	4	8	*0.002*	− 0.489
High grade	30	25	5		
Tumor stage^d^					
pT1-pT2	13	9	4	*0.040*	− 0.419
pT3-pT4	12	12	0		
RBBP8 methylation					
Low	11	5	6	*0.020*	− 0.564
High	7	7	0		

Significant *p* values are in italics

^a^Only patients with primary bladder cancer were included

^b^Score (IRS) according to Remmele and Stegner [42]

^c^Fisher’s exact test

^d^According to WHO 2004 classification

### *RBBP8* methylation is specifically detectable in urine samples derived from bladder cancer patients

**Fig. 6:**
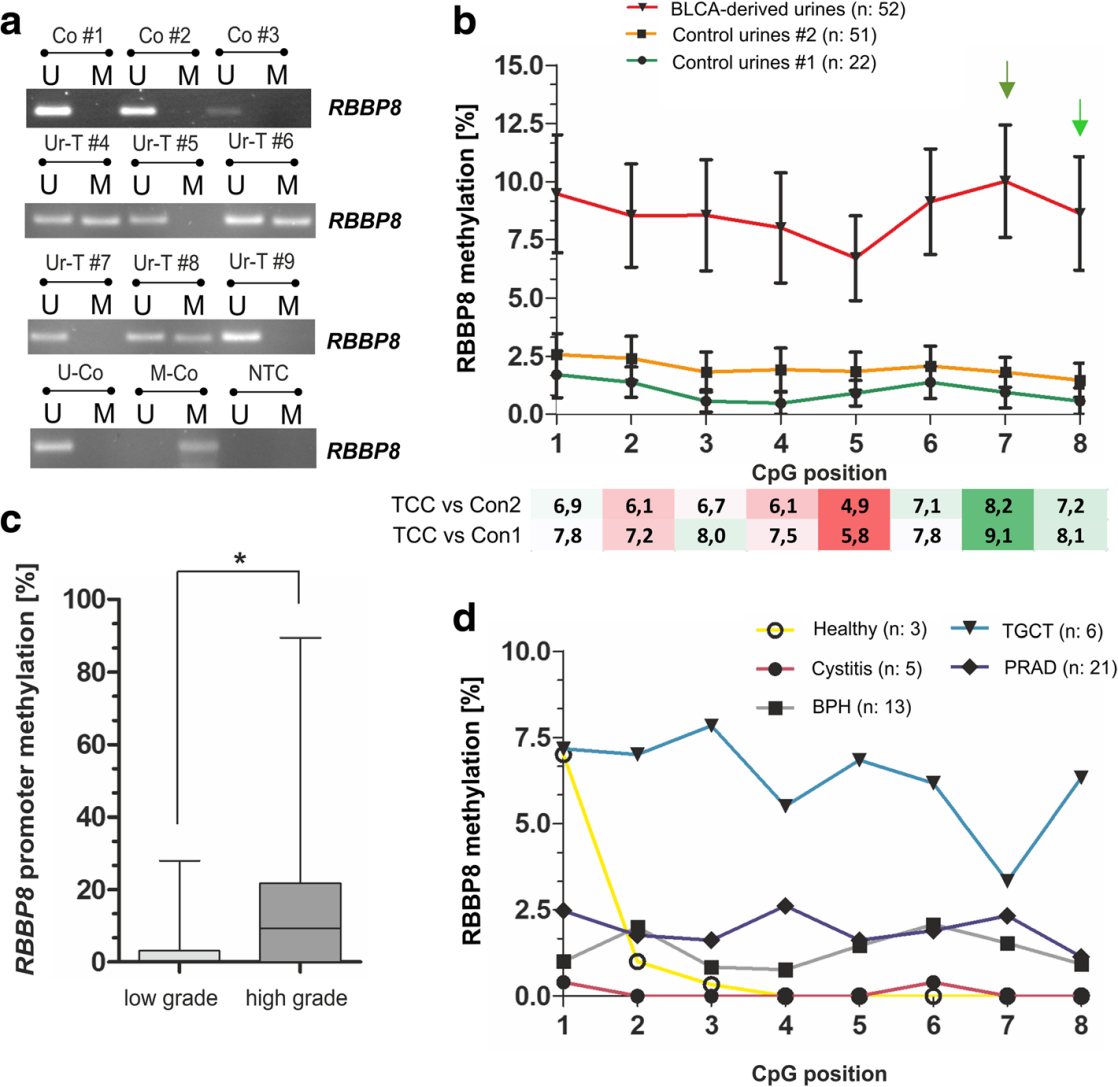
*RBBP8* promoter methylation is detectable in urines from bladder cancer patients. **a** Representative MSP analysis shows the *RBBP8* promoter methylation status of human urine samples derived from healthy controls (Co) and bladder tumors (Ur-T). Band labels with U and M represent an unmethylated and methylated DNA locus. Bisulfite-converted unmethylated, genomic (U-co), and polymethylated, genomic (M-co) DNA were used as positive controls. NTC, non-template control. **b** Upper graph: *RBBP8* mean methylation values of analyzed CpG sites (1 to 8) in cancerous bladder diseases (BLCA-derived) and two control cohorts (benign: control urines #1 and malignant: control urines #2) using pyrosequencing*.* Lower heatmap: Differences of *RBBP8* methylation between BLCA-derived urines and both control conditions (benign and malignant) highlighting GpG sites (#7 and #8) with the strongest impact for discrimination (see green arrows). **c** Box plot demonstrating a significant increase of *RBBP8* methylation in high-grade bladder tumors. Horizontal lines — grouped medians. Boxes — 25 to 75% quartiles. Vertical lines — range, peak, and minimum; *p < 0.05. **d** *RBBP8* mean methylation values of analyzed CpG sites (1 to 8) of controls classified by diseases*.* BPH, begin prostate hyperplasia; TGCT, testicular germ cell tumors; PRAD, prostate adenocarcinoma

As *RBBP8* is highly methylated in bladder tumor tissue, *RBBP8* methylated DNA might also be detectable in urine samples from bladder cancer patients. Therefore, urine sediments from bladder cancer patients (*n* = 22) and healthy controls (*n* = 10) were initially assessed for *RBBP8* methylation by MSP (for cohort characteristics see Additional file 10). DNA methylation of *RBBP8* was present in 11 out of 22 cancerous urine samples (50%) (Fig. 6a). In contrast, none of the control samples from healthy donors was tested positive for *RBBP8* methylation.

Next, we assessed *RBBP8* methylation using bisulfite-pyrosequencing in a collective (overall *n* = 103, see Table 5) comprising 52 BLCA-derived and 51 control urine samples. Control samples included inflammatory urological diseases and malignancies of the urological tract other than bladder cancer. Please note that the used control cohort which is enriched for urological malignancies does not reflect the epidemiological composition as found in populations, but served only to evaluate entity specificity of RBBP8. Quantification of *RBBP8* methylation allows specification of cutoff levels, which are necessary for an objective assessment of biomarker performance. Summarized data of the mean *RBBP8* methylation ratio for each CpG site of BLCA-derived and control urine set #1 (including healthy, inflammatory, and benign samples) and set #2 (including set #1 samples plus testicular germ cell tumors (TGCT) and prostate adenocarcinoma (PRAD) samples) is shown in Fig. 6b. *RBBP8* methylation was clearly increased, up to 88%, in urines from bladder cancer patients. As previously observed in primary bladder cancer, *RBBP8* promoter methylation detected in urines strongly correlates with high-grade tumors (Fig. 6c). This observation was statistically confirmed using a Fisher’s exact test (Table 6). Interestingly, *RBBP8* mean methylation of control urines from patients diagnosed with cystitis and benign prostatic hyperplasia (BPH) was nearly similar to healthy controls. In turn, a few urines from young patients with a testicular neoplasm (mostly seminomas) showed an increased mean *RBBP8* methylation (Fig. 6d).

**Table 5:** Clinicopathological parameters of urine samples analyzed in this study by pyrosequencing

Categorization	*n*	% analyzable
Controls:	51	
Age (median 69)		
≤ 69 years	28	54.9
> 69 years	23	45.1
Gender		
Male	48	94.1
Female	3	5.9
Diagnosis		
Disease-free	3	5.9
Cystitis	5	9.8
Benign prostatic hyperplasia	13	25.5
Prostate cancer	21	41.2
Testicular tumor	6	11.8
Other	3	5.9
BLCA asscociated^a^	52	
Age (median 71)		
≤ 71 years	27	51.9
> 71 years	25	48.1
Gender		
Male	42	80.8
Female	10	19.2
Histological tumor grade^b^		
Low grade	19	36.5
High grade	30	57.7
NA	3	5.8
Tumor stage^b^		
pTa	21	40.4
pTis	3	5.8
pT1	11	21.2
pT2	8	15.4
pT3	7	13.5
pT4	2	3.8

^a^Only urine samples of patients preoperatively diagnosed with primary bladder cancer (UC, without any other malignancy) were included

^b^According to WHO 2004 classification

**Table 6:** Clinicopathological parameters in relation to *RBBP8* promoter methylation in BLCA associated urine samples

	*RBBP8* promoter methylation^b^			
	*n* ^a^	Low	High	*p* value^c^
Parameter:				
Gender				
Male	42	31	11	0.687
Female	10	8	2	
Histological tumor grade				
Low grade	19	17	2	*0.046*
High grade	30	19	11	
Tumor stage				
pTa	21	19	2	*0.021*
pT1-pT4	28	17	11	
pN status				
Negative	5	3	2	0.931
Positive	8	5	3	

Significant *p* values are in italics

^a^Only patients with primary bladder cancer were included

^b^RBBP8 methylation was dichotomized at the Q75 (based on pyrosequencing)

^c^Fisher’s exact test

**Fig. 7:**
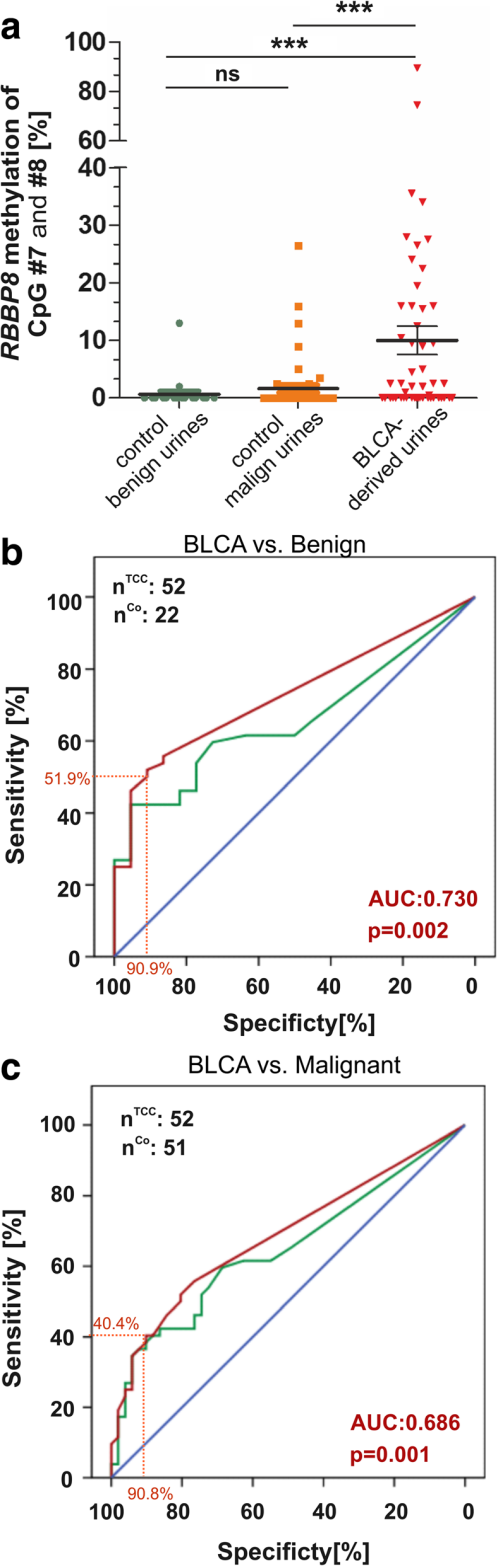
Biomarker performance of *RBBP8* methylation based on a non-invasive approach. **a** *RBBP8* methylation enables significant discrimination of cancerous bladder diseases from two control conditions (benign and malignant) using urine samples. The scatterplot shows the mean methylation values of the CpG sites #7 and #8; ns, not significant; ***p < 0.0001. **b** to **c** ROC curve analysis illustrating RBBP8 single-biomarker performance based on all analyzed CpG sites (green curve) and CpG site #7 and #8 (red curve). **b** ROC curve in benign disease controls. Red curve (CpG #7 and #8): the cutoff value of 1.25% methylation was defined for positive detection of disease; in that case, *RBBP8* methylation achieved a specificity of 90.9% and a sensitivity of 51.9%. Area under the curve (AUC) 0.730 (95% CI, 0.616 to 0.844), *p* = 0.002. **c** ROC curve in malignant (prostate and testicular cancer) disease controls. Red curve (CpG #7 and #8): the cutoff value of 4.00% methylation was defined for positive detection of disease; in that case, *RBBP8* methylation achieved a specificity of 90.2% and a sensitivity of 40.4%. Area under the curve (AUC) 0.686 (95% CI, 0.583 to 0.789), *p* = 0.001

In order to evaluate its diagnostic potential, we performed receiver operating characteristics (ROC) curve analysis to calculate the optimal cutoff value for *RBBP8* as single marker, with a high specificity. ROC curve statistics were performed based on the two CpG sites with the strongest discrimination impact; the methylation ratio between cancerous BLCA urines and both the benign and malignant control set revealed strongest differences for CpG number #7 and #8 (see Fig. 6b). There was no significant difference between benign and malignant control urine samples, while *RBBP8* methylation based on “best CpG sites” was highly significantly increased in BLCA-derived urines (Fig. 7a). Accordingly, *RBBP8* methylation significantly discriminates between bladder cancer patients and non-malignant patients with a sensitivity of 51.9% and a specificity of 90.9% (AUC 0.730, 95% CI 0.616–0.844) (Fig. 7b). In case of 100% specificity, *RBBP8* methylation as a single biomarker still showed a true positive rate of 25% (see Table 7). The control cohort consisted of patients with an inflammatory and benign diagnosis (benign control set). Additionally, the single *RBBP8* marker was able to distinguish bladder cancer patients from patients with neoplasms of another urological origin (malignant control set) with high specificity (90.8%, AUC 0.686, 95% CI 0.583–0.789). Sensitivity was however reduced to 40.4% (Fig. 7c). Thus, beyond a possible prognostic or predictive relevance of RBBP8 loss in human cancers, its promoter methylation may be suitable for diagnostic or monitoring applications in bladder cancer. Since a *RBBP8* methylation frequency of approximately 40% is found in bladder cancer, sensitivity is limited. Consequently, a combined application as part of a biomarker panel will be necessary to increase the true positive rate of detection.

**Table 7:** RBBP8 biomarker performance

Cutoff	Specificity (%)	Sensitivity (%)	AUC	*p* value	Control group
*RBBP8* CpG #7 and #8					
14.25	100	25.0	0.730	0.002	Benign
1.25	90.9	51.9			
14.25	96.1	25.0	0.686	0.001	Malignant
4.00	90.2	40.4			
*RBBP8* CpG all					
11.75	100	26.9	0.660	0.030	Benign
0.69	77.3	51.9			
11.75	96.1	26.9	0.649	0.009	Malignant
3.94	90.2	38.5			

## Discussion

Dysfunction of DNA damage repair (DDR) genes in human cancer is common and, so far, mostly considered to be triggered by genetic alterations [44]. Yet, in 2015, Gao et al. proposed a notable clinical value of epigenetic alterations in distinct DDR genes in human cancer [12]. To our best knowledge, our study provides for the first time a systematic analysis of the epigenetic configuration of DNA repair genes involved in the DNA damage response across human cancer entities. As DDR genes are known to be important targets for novel therapeutic strategies [8–11] the genome-wide epigenetic configuration within promoter regions of DDR genes can be used to identify novel targets of epigenetic alterations. These targets may hold clues for non-invasive biomarker applications with predictive impact.

In the presented study, tumor-specific epigenetic deregulation of DDR genes was found to be a common event in human cancer. Statistical analysis of genome-wide 450K methylation array data identified 39 out of 177 DDR genes, whose promoter regions close to the TSS were hypermethylated in one or several tumor entities. Most tumor entities were characterized by hypermethylation of distinct DDR genes. Among others, polymerase delta (*POLD1*) methylation was enriched in 24 out of 32 different tumor types, implying an essential role in DNA repair or tumor suppression independent of tumor entity. This includes COAD and LIHC, i.e., entities which have been associated with increased somatic mutation rates [4]. Unrepaired DNA damage is a major source of potential mutagenic lesions driving tumorigenesis, and loss of DNA repair pathways is thought to accelerate the accumulation rate of additional mutations by 100 to 1000 times [45]. The *POLD1* gene is highly conserved and encodes for the p125 subunit which provides the essential catalytic activity of polymerase δ (Polδ), mediating a key role in genome stability [46]. This raises the question whether or how methylation of the identified DDR genes may help to establish mutator phenotypes affecting the prevalence of somatic mutations in distinct tumor types. Weisenberger et al. previously revealed a close association between the CpG island methylator phenotype (CIMP), microsatellite instability, and increased BRAF mutation in COAD [47]. Moreover, sporadic cases of mismatch repair deficiency occur almost exclusively as a consequence of CIMP-associated *MLH1* methylation, a DNA repair gene involved in MMR and microsatellite instability [48], whose hypermethylation close to the TSS was confirmed for COAD in the presented study.

Beyond, our findings may provide novel implications for therapies, as DNA damage repair dysregulation sensitizes cancer cells for DNA damaging agents [12]. Novel biomarkers with a clear prognostic or predictive impact are urgently required to identify those patients likely to respond favorably to such therapy, for instance, in BLCA. There is no significant progress in systemic therapy for muscle-invasive bladder cancer (MIBC) since decades [21, 49] with the exception of immunotherapeutic approaches like PDL-1 blocking antibodies [50]. Due to the lack of biomarkers [51], disease management of BLCA and hence the selection of an adequate therapy are difficult and often insufficient [52]. Here, we show that 2%, i.e., 6 out of 368 bladder patients, showed O6-methylguanine-DNA methylftransferase (*MGMT*) promoter hypermethylation which is in line with previous reports [53–55]. The *MGMT* promoter status has been shown to predict response to alkylating agents in glioblastoma patients [13, 14], and in times of personalized and precision medicine even such low case numbers could be of clinical significance. Furthermore, the excision repair cross-complementing group 1 (*ERCC1)* gene, encoding for a known key enzyme of the nucleotide excision repair (NER) pathway [56], was found frequently methylated in BLCA, up to 17%. Accumulating studies propose ERCC1 protein expression as a prognostic and predictive biomarker for platinum resistance in various tumor entities [37, 57] including bladder cancer [36, 58]. In BLCA, for instance, ERCC1-negative tumors could benefit from adjuvant chemotherapy combining gemcitabine and cisplatin [36], but its predictive impact remains controversial [59]. Future studies addressing the potential clinical impact of *ERCC1* and the relation between its promoter methylation and gene silencing are important. Such a functional consequence would offer the development of predictive biomarkers based on a stable analyte (i.e., DNAm) which can be efficiently detected in bodily liquids like urine, in analogy to the here exemplarily identified and validated DDR lead candidate gene *RBBP8/CtiP* in BLCA.

*RBBP8*/*CtiP* has a proven role in homologous recombination-mediated DNA double-strand break repair (HR and NHEJ pathway) [30, 32, 33], impairment of which reduces DNA repair fidelity and may promote genome instability [34, 60] also in urothelial carcinomas. Already in 2006, genomic analysis described bladder cancer as significantly impaired in DNA repair [20]. Here, we demonstrate for the first time that *RBBP8* is almost exclusively methylated in primary BLCA, suggesting the development of methylation phenotypes in a distinct molecular context. *RBBP8* methylation was confirmed to be tumor-specific in up to 45% of analyzed BLCA patients. A close association between *RBBP8* methylation and its gene expression in primary tumors as well as after demethylation treatment in vitro indicate that *RBBP8* methylation could be responsible for its gene inactivation. Hence, it seems unlikely that entity-specific *RBBP8* methylation is a silent passenger event without relevance, and we propose loss, or at least reduction, of RBBP8 mediated DNA repair function in BLCA. Interestingly, *RBBP8* methylation correlates with a favorable prognosis in the BLCA TCGA dataset. Functionally, the RBBP8 protein is known to interact with BRCA1 [61], guiding HR by recruiting Dna2 to damage sites, thus ensuring a robust DSB resection necessary for efficient homologous recombination [30]. Previously, it has been shown that BRCA1/2 deficiency results in cellular sensitivity to chemotherapy [9, 62, 63]. For example, *BRCA1* methylation has been revealed to predict significantly higher response rates to cisplatin treatment in breast and ovarian cancer patients [17], which is also traditionally used as the first-line agent in bladder cancer disease management [21]. Cisplatin-induced inter-strand adducts can lead to DNA lesions (double-strand breaks) which are regularly removed by the machinery of HR [64]. Unrepaired inter-strand crosslinks as consequence of reduced RBBP8 function may thereof sensitize tumor cells to chemotherapy treatment as well. Considering that a high *RBBP8* methylation frequency correlates with increased survival of BLCA patients, *RBBP8* methylation might be a potential biomarker for better therapy response in such subsets of patients; however, further investigation is necessary to validate this hypothetical notion. Nevertheless, accumulating studies already functionally demonstrated the involvement of RBBP8 on sensitizing breast [27] and ovarian cancer cells [26] for PARP inhibitor treatment in a similar way as *BRCA1* mutations, thus unveiling novel therapeutic options for a significant subset of patients which may benefit from such approach.

The field of liquid biopsy is rapidly evolving [65] and shows that biomarkers can be detected in biological fluids like blood or urine, offering an easy and non-invasive application in diagnosis of and therapy prediction in cancer. Application of liquid biopsy tools are of importance in bladder cancer, as its clinical management remains challenging due to its high recurrence rate (up to 70%) [66] in the first 2 years after diagnosis, requiring a lifetime of surveillance, i.e., BLCA patients undergo multiple invasive procedures. One of these methods is the cystoscopy, representing the gold standard test for the detection of bladder cancer, achieving an operator-dependent sensitivity over 90%. Cystoscopy is however associated with significant discomfort, possible risk of infection, and high costs [67, 68]. Current guidelines recommend the application of non-invasive urine cytology testing complementary to cystoscopic assessment. Nevertheless, cytology is characterized by poor sensitivity, especially for low-grade and low-stage tumors like CIS [69]. In addition, previously developed non-invasive diagnostic tools, such as the “BladderCheck (point-of-care) Test” based on the protein marker NMP22, either lack sensitivity or specificity [69]. Urine-based tests assessing aberrant DNA methylation are emerging as a potential tool for cancer detection [6]**,** and various studies described the identification of novel DNA methylation-based biomarkers. Nevertheless, although both multiple biomarker tests and single-biomarker candidates like *Vimentin* [70] were able to provide prognostic information, they did not show any predictive impact. In addition, specificity across tumor entities is disregarded in most studies. Here, we demonstrate that *RBBP8* hypermethylation was accessible through a non-invasive urine test. Using *RBBP8* as a methylation biomarker, BLCA could be detected with a 25% sensitivity (at maximal specificity of 100%) in urines from BLCA patients, i.e., enabling to distinguish early cancerous (CIS), high-grade urothelial, and even papillary subtype tumors, which are difficult to detect by urine cytology, from non-cancerous and benign ones. At 90.9% specificity, the sensitivity of *RBBP8* was even increased up to 51.9*%*. Importantly, in comparison to a control urine cohort including urological malignancies like prostate and testicular cancer, we were still able to detect over 40% of primary bladder tumors with high specificity. Hence, we provide a novel biomarker candidate, i.e., *RBBP8*, which has previously been shown to sensitize tumor cells for PARP1 inhibitors and which may improve entity-specificity for monitoring, early detection, and clinical management of bladder cancer in a urine biomarker panel.

## Conclusions

In the current study, we systemically screened for epigenetic configuration of DNA repair genes involved in the DNA damage response (DDR) across 32 human cancer entities and identified 39 methylated DDR genes which may be important targets for novel therapeutic strategies. This includes known epigenetic silenced repair genes like *MGMT*, *MLH1*, and *ERCC1* but also novel targets of epigenetic dysregulation like *RBBP8/CtiP*, which has a proven role in homologous recombination-mediated DNA double-strand break repair and is further known to sensitize cancer cells for PARP1 inhibitors. *RBBP8* was almost exclusively hypermethylated in bladder cancers and was detectable by a non-invasive approach in urines from bladder cancer patients. Hence, *RBBP8* might serve as a complementary biomarker of high specificity that can be accessed through a urine test. Further investigation of mechanisms involving RBBP8 may deliver novel therapeutic options for bladder cancer patients, finally highlighting RBBP8 as a predictive biomarker as well.

## Methods

### Reagents

The DNA methyltransferase (DNMT) inhibitor 5-aza-2′-deoxycytidine (decitabine (DAC); 1 mM in phosphate-buffered saline (PBS)) and the histone deacetylase inhibitor trichostatin A (TSA; 1 mM in PBS) were obtained from Sigma-Aldrich. Drugs were stored in aliquots at − 80 °C.

### Cell lines

Unless stated otherwise, cell lines were obtained from the American Type Culture Collection (ATCC, Rockville, MD) and cultured as recommended by the vendor. ATCC provides molecular authentication in support of their collection through their genomics, immunology, and proteomic cores, as described, by using DNA barcoding and species identification, quantitative gene expression, and transcriptomic analyses. Cells were tested negative for mycoplasma infection before and after experiments. The 37 used cell lines were UROtsa (normal bladder urothelium), RT4, RT112, EJ28, J82 (bladder cancer), RWPE-1 (normal prostate epithelium), LNCaP, PC3, DU145 (prostate cancer), SKRC1, SKRC10, SKRC52, SKRC59 (clear cell renal cell carcinoma), HCT-116, RKO, CACO2, COLO205, HT29, SW480, CACO320 (colorectal cancer), MCF10A, MCF12A, MCF7, T-47D, ZR75-1, BT474, SKBR3, MDA-MB-231, MDA-MB-436, MDA-MB-468, UACC3199, HCC1937, BT20 (breast cancer), A549, H157, and H2170 (lung cancer).

### Formalin-fixed paraffin-embedded tissue specimens

Tissues of primary bladder tumors and normal urothelium were obtained from the archives of the Institute of Pathology (RWTH Aachen University). The anonymized and retrospective study was approved by the local Ethics Committee (EK 122/04, 173/06, and 206/09). For cohort characteristics of analyzed samples, see Additional file 8. Only manually microdissected samples were used for *RBBP8* methylation analysis. RBBP8 protein expression was evaluated using whole tissue sections or tissue microarrays with representative tissue cores of 1.5 mm diameter.

### Human urine samples

Voided urine samples from patients diagnosed with a primary bladder tumor (*n* = 52) were used to assess biomarker performance. Urine samples of patients with a known second malignancy such as prostate cancer were excluded from the study. Urines from healthy donors (*n* = 13) and samples derived from patients with 5 inflammatory (chronic cystitis), 13 benign (benign prostate hyperplasia), and 27 urological malignant diseases (testicular tumors and prostate cancer) served as controls. For cohort characteristics, see Table 5 and Additional file 10. All urine specimens were obtained from the RWTH centralized biomaterial bank (RWTH cBMB) and the Technical University of Munich. All patients gave written consent for retention and analysis of their samples according to local Institutional Review Board (IRB)-approved protocols of the Medical Faculty of RWTH Aachen University and the Technical University of Munich. For each sample, 20 ml of (morning) urines were centrifuged for 10 min at 2000×*g*, and sediments were stored at − 80 °C.

### Genome-wide methylation analysis using TCGA data sets

#### Data retrieval

Infinium HumanMethylation450 BeadChip data (level 2) and RNASeqV2 data (level 3) of the tumor and normal tissue samples were obtained from the TCGA data portal [1–4]. Samples with any annotations reported via the TCGA annotation portal were excluded from the analyses. The *β* value of methylation was calculated according to Bibikova et al. [71] for all CpG probes with a detection *p* value ≤ 0.05. Probes with a detected *p* value above the threshold were classified as unmeasured. Multiple measurements per tumor were averaged after log-transformation of expression data and transformation of methylation data to *M* values [72]. In total, 7819 tumors and 659 normal samples were included in the differential CpG methylation analysis (Table 1). Five thousand one hundred ninety-one tumor samples were included in the correlation analysis of gene expression and CpG methylation. A summary of the TCGA samples that used both datasets can be found in Additional file 11.

#### Identification of CpG sites close to TSS of DDR genes

A list of 177 DNA damage repair genes was used for genome-wide analysis of which none was located on the sex chromosomes (Additional file 12). Information on gene location and transcription start sites (TSS) was obtained from the Ensembl database. For each gene, CpG probes (probe = CpG site) located 2000 bp downstream to 500 bp upstream of a reported TSS were determined.

#### Definition of probe sets near a TSS

An exploratory data analysis was performed to identify genomic regions with tumor-specific methylation, i.e., showing unmethylated CpGs in available normal tissue samples. In brief, clusters of TSS were identified (hierarchical clustering with single linkage; cut at a height of 2000 bp). Available methylation data from normal tissues were then visualized (see Additional file 13) in the regions surrounding the TSS clusters. Probes of CpGs in continuously unmethylated (*β* < 0.25) regions were grouped together as a set. Based on both the entity-specific patterns of methylation in tumor tissues (see Additional file 13) and the correlation coefficients between CpG sites (see Additional file 13), probe sets were further classified to define CpG (probe) sets with a putative regulatory impact within promoter regions of DDR genes. This approach led to the identification of one to four CpG (probe) sets for most candidate genes (170 out of 177). The majority (140 out of 170) of these genes showed only one CpG set, while for some genes, two (20 out of 170), three (2 out of 170) or four (1 out of 170) sets were considered. The detailed definition of used CpG probe sets for DDR genes can be found in the supplementary data (Additional file 14).

#### Determination of entity-specific frequency of hypermethylation

Given the definition of CpG probe sets as promoter regions potentially involved in gene regulation of the corresponding DDR gene, frequency of hypermethylation in tumor tissues from different entities was calculated for each gene. With respect to known characteristics of used tumor samples (i.e., the contamination with stromal cells (e.g., fibroblasts) in up to 40% of TCGA specimens; the copy number variations or whole genome duplications (e.g., [73]); and lastly, the prevalence of subclones) which could affect tumor-associated allele frequencies, a stepwise prioritization strategy with adjusted *β* value thresholds was conducted.

A CpG set specific methylation value was determined by the calculation of the median methylation level for each sample. Secondly, as the *M* value is more statistically valid for the differential analysis of methylation levels [72], a probe set specific (∆*β*_co_) cutoff for the tumor samples was derived jointly from all available normal samples after transformation to *M* values as the arithmetic mean plus three standard deviations. To further ensure robust confirmation of these findings by alternative methods such as pyrosequencing of sodium bisulfite converted DNA (e.g., [74, 75]), we increased this cutoff by a *β* value of 0.1 (see Additional file 14), hence defining minimum *β* value differences (∆*β*_co_) of 0.1–0.2 between normal and tumor samples which is in line with previous studies (e.g., [76]). In practice, a set specific *β* value above approximately 0.25 (90% CI 0.20–0.35) was considered to indicate hypermethylation of TSS regions in tumor samples. The fraction of samples with a hypermethylation event was calculated for each tumor entity separately.

### In vitro genomic DNA demethylating treatment

For gene re-expression experiments, cells were seeded at 2.5 × 10^4^ cells/cm^2^ in 6-well plates. After overnight attachment, the DNMT inhibitor DAC was added and renewed every 24 h. For a combined treatment with a histone deacetylase inhibitor, TSA was added at 300 nM to cells 16 h prior to RNA extraction. After 72 h, cells were harvested, and RNA was extracted.

### Nucleic acid extraction from cell lines, FFPE tissues, and urines

Sections of archival formalin-fixed paraffin-embedded (FFPE) tissue samples were microdissected, when necessary, deparaffinized in xylene, and re-hydrated in a decreasing alcohol series prior to nucleic acid extraction. Genomic DNA of bladder samples was isolated using the QIAamp® DNA Mini kit (Qiagen, Hilden, Germany). Total RNA from cell lines was extracted using the TRIzol® (Life Technologies) method according to the manufacturers’ instructions.

Sediments of urine samples stored at − 80 °C were subjected to DNA extraction using the ZR Urine DNA Isolation Kit (ZR, Zymo Research, Orange, Ca, USA) following the manufacturer’s instructions. The DNA yield (ng/ml urine) and purity (A_260_/A_280_) were determined by using the NanoDrop (Thermo Fisher Scientific, Waltham, MA, USA). Only extractions from urines with a minimal amount of 100 ng genomic DNA and a ratio of > 1.5 were finally used for MSP and pyrosequencing analyses. Note that the extracted DNA derived from FFPE tissue samples are known to be more fragmented and altered compared, for instance, to DNA from urines. Therefore, results like methylation frequencies may slightly differ between both materials.

### Bisulfite conversion and methylation-specific PCR

Five hundred nanogram of the genomic DNA was bisulfite-converted for 16 h using the EZ DNA Methylation™ kit (Zymo Research, Freiburg, Germany) according to the manufacturer’s instructions. Bisulfite-converted DNA was eluted in 20 μl of TRIS-EDTA buffer. MSP was performed as previously described [77]. In brief, 150 to 500 ng of converted DNA was amplified using MSP primers (designed by using MethyPrimer software [78]; for primers, see Additional file 15) that specifically met the following criteria: (1) In order to validate TCGA datasets, MSP primers recognizing either the unmethylated or methylated *RBBP8* gene sequence, covered CpG sites also used in the TCGA data analysis. (2) To reveal whether the epigenetic alteration could be responsible for *RBBP8* gene inactivation, which is a requirement for a causative impact on therapeutic responses, designed MSP primers annealed closely to the TSS (core promoter region). The EpiTect® PCR Control DNA Set (Qiagen) was used as positive controls for unmethylated and methylated DNA. Amplified products were visualized on 2% agarose gels (Biozym, Hessian Oldendorf, Germany) containing ethidium bromide (AppliChem, Darmstadt, Germany) and illuminated under UV light.

### Bisulfite-pyrosequencing

In order to assess *RBBP8* methylation in detail, pyrosequencing of eight CpG sites was performed that are located in the *RBBP8* promoter area close to the transcription start site. The *RBBP8* pyrosequencing assay was designed by using the Pyromark Assay Design Software (Qiagen), and all primers are listed in Additional file 16. Initially, a 136-bp fragment of the *RBBP8* promoter region including CpG sites of both the complete MSP amplicon and the TCGA analysis was amplified by using the PyroMark PCR Kit (Qiagen). Degenerate PCR primers assured unbiased DNA amplification independently of the methylation status. A 73-pb region of the PCR amplicon, referred to as the sequence of interest, was subsequently sequenced covering eight CpG sites. Methylation ratio for each CpG of the *RBBP8* promoter was quantified based on the PyroMark96 ID device and the PyroGoldSQA reagent Kit (Qiagen) as previously specified [79].

### Reverse transcription and quantitative RT-PCR

One microgram of the total RNA was reverse transcribed using the Reverse Transcription System (Promega, Mannheim, Germany). qRT-PCR was accomplished on an iQ5 Real-Time PCR Detection System (Bio-Rad, München, Germany) together with the iQ™ SYBR® Green Supermix (Bio-Rad) including 5 μM of intron-spanning oligonucleotide primers (see Additional file 17) and 20 ng of cDNA in a 20 μl reaction volume. The cycling profile was 95 °C for 10 min, followed by 40 cycles of 95 °C for 30 s and 60 °C for 60 s. All reactions were run in triplicate, and post-amplification melting curve analyses assessed the product specificity. Gene expression relative to the housekeeping gene glyceraldehyde 3-phosphate dehydrogenase (*GAPDH*) was calculated by the ΔΔ*C*_T_ method [80].

### RBBP8 immunohistochemistry

Immunohistochemical staining of RBBP8 protein was performed after heat-induced antigen retrieval (EnVision™ FLEX Target Retrieval Solution, Low pH, K8005, DAKO PT-Link, DAKO, Hamburg, Germany) according to the manufacturer’s protocols. Primary anti-RBBP8 antibody (dilution 1:200) (Atlas Antibodies, HPA039890, Sigma-Aldrich, Germany) was linked with DAKO EnVision™FLEX system and visualized with DAKO Liquid DAB Substrate Chromogen System in a DAKO Autostainer plus (K8024, K3468, DAKO). RBBP8 protein staining was quantified by a pathologist using an adapted immunoreactive scoring system (IRS) according to Remmele and Stegner [42].

### Statistical analyses

Data analyses and statistical correlations of TCGA datasets were performed using R Statistical Software version 3.2.0 [81] (R Foundation for Statistical Computing, Vienna, Austria). The parameters of beta distributions were estimated by the maximum likelihood estimator implemented in the *RPMM* package [82]. Two-sided *p* values less than 0.05 were considered significant. In order to compare two groups, the non-parametric Mann-Whitney *U* test was implemented, whereas in case of more than two groups, the Dunn’s multiple comparison test was used. Statistical associations between clinicopathological and molecular factors were determined by Fisher’s exact test using SPSS software version 22.0 (SPSS Inc., Chicago, USA). Survival curves for overall survival (OS) were calculated using the Kaplan-Meier method with log-rank statistics. OS was measured from surgery until death and was censored for patients alive without evidence of death at the last follow-up date. Multivariate Cox regression analysis was performed to test for an independent prognostic value of *RBBP8* methylation. Receiver operating characteristics (ROC) curves were calculated to assess biomarker performance of *RBBP8* methylation in urine samples [83].

## Additional files

Additional file 1: Schematic map of the underlying study design comprising the discovery and the validation step. (DOCX 146 kb)

Additional file 2: Detailed frequency of DNAm of *BRCA1*, *MGMT*, and *ERCC1* across cancer entities and corresponding normal tissues. (DOCX 296 kb)

Additional file 3: Heatmap of tumor-specific DNAm of DDR genes across cancer entities. (DOCX 345 kb)

Additional file 4: Graphs illustrating the correlation between DDR gene hypermethylation and their gene expression tumor entities. (DOCX 358 kb)

Additional file 5: This table illustrates the clinicopathological parameters of 405 bladder cancer specimens of the TCGA network analyzed in this study. (DOC 48 kb)

Additional file 6: This table summarizes the results from the multivariate Cox regression analysis including all factors influencing overall survival. (DOC 34 kb)

Additional file 7: This table lists the *RBBP8* methylation in human cell lines analyzed by MSP. (DOC 52 kb)

Additional file 8: This table shows the results of bivariate correlation statistics (Fisher exact test) between clinicopathological parameters and *RBBP8* methylation. (DOC 49 kb)

Additional file 9: A table is shown characterizing 42 bladder cancer specimens analyzed in this study by IHC. (DOC 46 kb)

Additional file 10: This table summarizes the clinicopathological parameters of urine samples analyzed in this study by MSP. (DOC 48 kb)

Additional file 11: List of 177 DDR genes analyzed in this study. (XLS 1076 kb)

Additional file 12: This dataset illustrates a summary of the TCGA samples used in this study. (XLS 27 kb)

Additional file 13: Illustrating the definition of probe sets near a TSS using three different genes (*RBBP8*, *MGMT*, and *LIG4*). (DOCX 1053 kb)

Additional file 14: This dataset summarizes the beta values according to a defined cutoff of 0.1. (XLS 62 kb)

Additional file 15: This table shows the sequences of all primers and conditions used in this study for MSP analysis. (DOC 33 kb)

Additional file 16: This table lists the primer sequences for bisulfite-pyrosequencing of the *RBBP8* promoter region. (DOC 32 kb)

Additional file 17: Sequences of all primers and conditions for qPCR analysis are summarized in a table. (DOC 32 kb)

## Abbreviations

ATCC: American Type Culture Collection
AUC: Area under curve
BER: Base excision repair
BLCA: Bladder cancer
BPH: Benign prostatic hyperplasia
BRCA1/2: Breast cancer 1/2, early onset
cBMB: Centralized biomaterial bank
cDNA: Copy number desoxyribonucleic acid
CIMP: CpG island methylator phenotype
CpG: 5′-deoxycytidine-phosphate-deoxyguanosine-3′
CtiP: CtBP-interacting protein
DAC: Decitabine
DDR: DNA damage repair
DNA: Deoxyribonucleic acid
DNAm: DNA methylation
DNMT: DNA methyltransferase
DSB: Double-strand break
EK: Ethics committee
ERCC1: Excision repair cross-complementing group 1
FC: Fold change
FFPE: Formalin-fixed paraffin-embedded
GAPDH: Glyeradehyde 3-phosphate dehydrogenase
HR: Homologous recombination
IHC: Immunohistochemistry
IRS: Immunoreactive score
MGMT: O6-methylguanine-DNA methyltransferase
MIBC: Muscle-invasive bladder cancer
MLH1: MutL homolog 1
MMR: Mismatch repair
mRNA: Messenger ribonucleic acid
MSP: Methylation-specific PCR
n: Number
NER: Nucleotide excision repair
NHEJ: Non-homologous end-joining
OS: Overall survival
PARP: Poly (ADP-ribose) polymerase
PBS: Phosphate-buffered saline
PCR: Polymerase chain reaction
POLD1: Polymerase delta 1
qPCR: Quantitative PCR
ROC: Receiver operating characteristic
RWTH: Rheinisch-Westfälisch Technische Hochschule
SCC: Squamous cell carcinoma
s.d.: Standard deviation
s.e.m.: Standard error of the margin
TCGA: The Cancer Genome Atlas
TSA: Trichostatin A
TSS: Transcription start site
USA: United States of America
