# Supplementary material for: Promoter methylation of DNA damage repair (DDR) genes in human tumor entities: RBBP8/CtIP is almost exclusively methylated in bladder cancer
Source: Clin Epigenetics. 2018 Feb 6;10:15. doi: 10.1186/s13148-018-0447-6 (PMC5802064; doi:10.1186/s13148-018-0447-6)
Supplement: Supplementary file 1 — Schematic map of the underlying study design comprising the discovery and the validation step. (DOCX 146 kb) [file 13148_2018_447_MOESM1_ESM.docx]

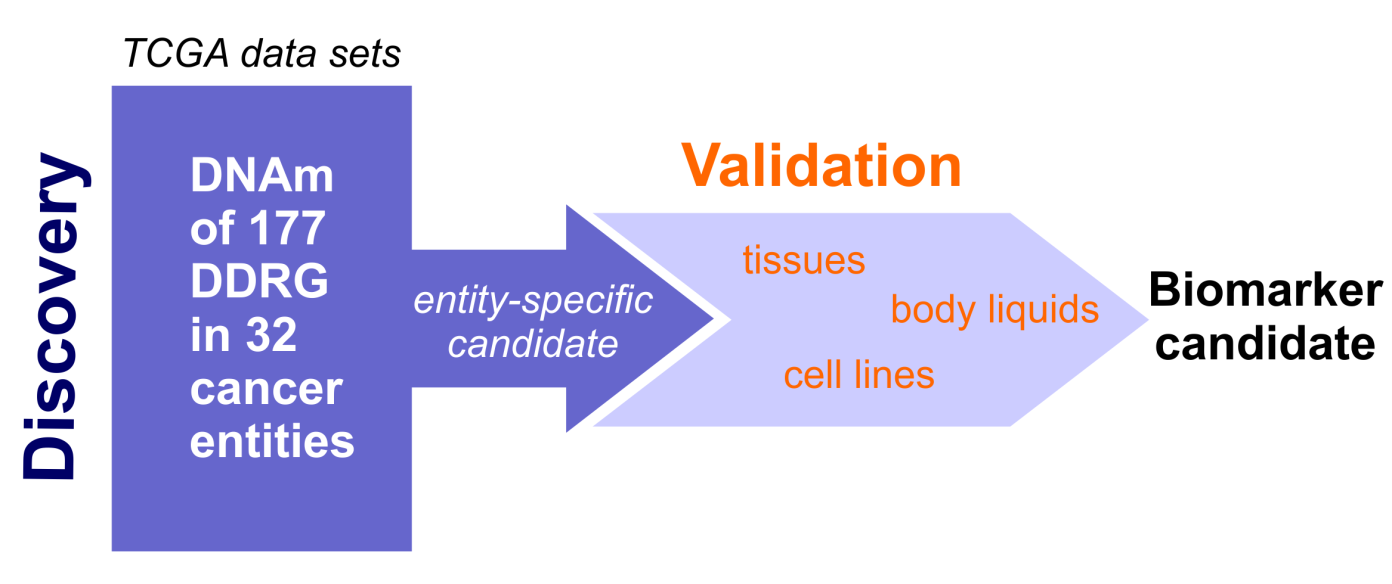


**Figure S1: Schematic map of the underlying study design:** Genome-wide DNA methylation (DNAm) analysis of promoter regions of DDR genes was performed to identify both co-existing unique DNAm patterns in tumor types and entity-specific targets of epigenetic deregulation. 1. Step: Discovery. Data sets of the Infinium HumanMethylation450 BeadChip platform of the TCGA project were used to determine tumor-specific, aberrant hypermethylation of promoter regions of 177 DDR genes in 32 different tumor entities. **Step 2: Validation.** Based on defined criteria candidate(s) were identified (entity-specificity, correlation between DNAm and mRNA expression, prognostic impact) and subsequently validated using primary tissue cohorts, cell lines and body liquids to assess potential biomarker performances for non-invasive detection approaches.
