# Supplementary material for: Promoter methylation of DNA damage repair (DDR) genes in human tumor entities: RBBP8/CtIP is almost exclusively methylated in bladder cancer
Source: Clin Epigenetics. 2018 Feb 6;10:15. doi: 10.1186/s13148-018-0447-6 (PMC5802064; doi:10.1186/s13148-018-0447-6)
Supplement: Supplementary file 2 — Detailed frequency of DNAm of BRCA1, MGMT, and ERCC1 across cancer entities and corresponding normal tissues. (DOCX 296 kb) [file 13148_2018_447_MOESM2_ESM.docx]

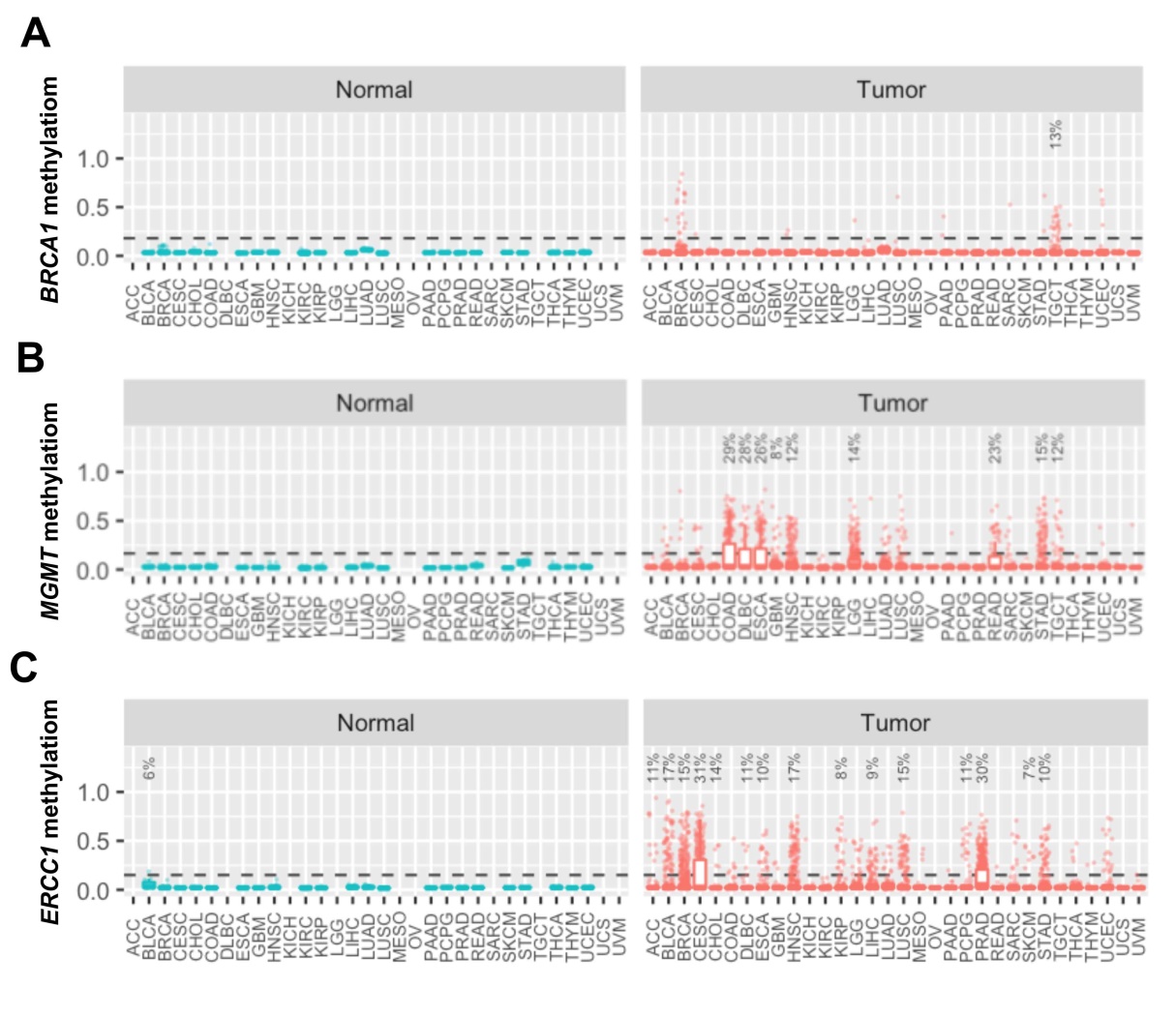


**Figure S2: Detailed frequency of DNAm of *BRCA1*, *MGMT* and *ERCC1* across tumor entities and corresponding normal tissues.** The β-values of all probes of promoter regions of *BRCA1* **(A)** *MGMT* **(B)** and *ERCC1* **(C)** were summarized by their median value, stratified by sample as well as tissue type and visualized as a boxplot.
