# Supplementary material for: Promoter methylation of DNA damage repair (DDR) genes in human tumor entities: RBBP8/CtIP is almost exclusively methylated in bladder cancer
Source: Clin Epigenetics. 2018 Feb 6;10:15. doi: 10.1186/s13148-018-0447-6 (PMC5802064; doi:10.1186/s13148-018-0447-6)
Supplement: Supplementary file 3 — Heatmap of tumor-specific DNAm of DDR genes across cancer entities. (DOCX 345 kb) [file 13148_2018_447_MOESM3_ESM.docx]

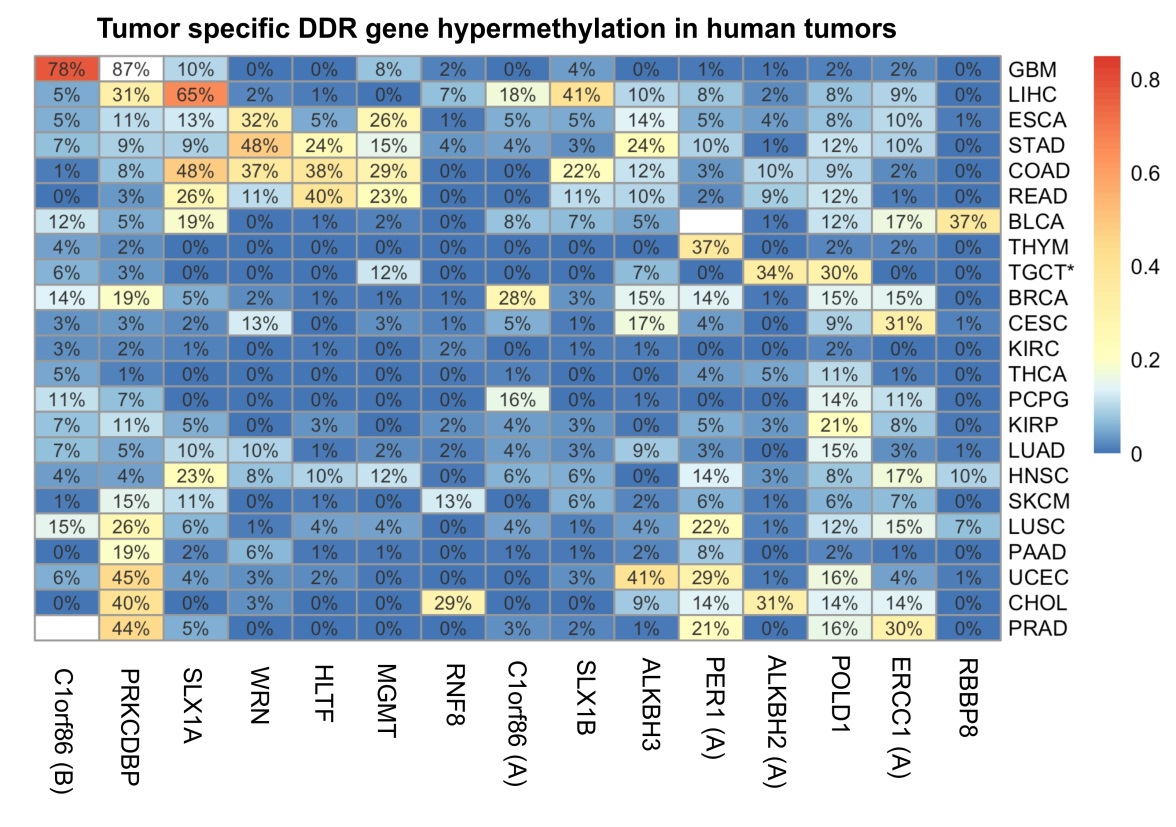


**Figure S3: Tumor specific DNAm of DDR genes across tumor entities.** Promoter methylation of DNA damage repair genes was present in 14 different tumor entities (*TGCT was included despite missing normal tissue samples). The heatmap illustrates the conducted analysis of the Infinium HumanMethylation450 BeadChip data available from the TCGA project (see Material and Methods for details). Only those genes (CpG groups close to TSS(s)) for which at least one tumor entity exhibited a hypermethylation of more than 15% of cases are shown. Fields for which corresponding normal tissues were hypermethylated in more than 15% are not shown (white). Fields were clustered hierarchical in both dimensions.
