# Supplementary material for: Promoter methylation of DNA damage repair (DDR) genes in human tumor entities: RBBP8/CtIP is almost exclusively methylated in bladder cancer
Source: Clin Epigenetics. 2018 Feb 6;10:15. doi: 10.1186/s13148-018-0447-6 (PMC5802064; doi:10.1186/s13148-018-0447-6)
Supplement: Supplementary file 4 — Graphs illustrating the correlation between DDR gene hypermethylation and their gene expression tumor entities. (DOCX 358 kb) [file 13148_2018_447_MOESM4_ESM.docx]

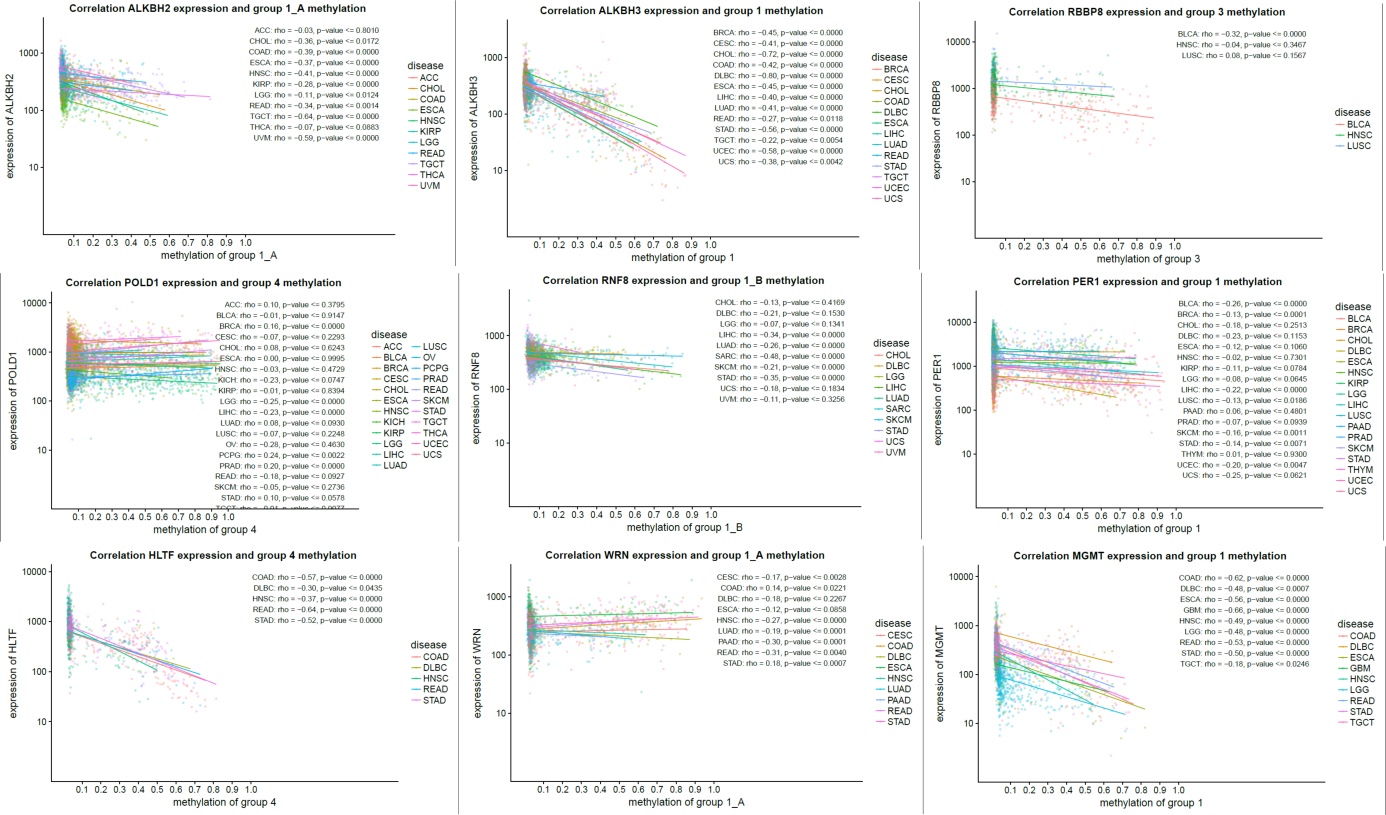


**Figure S4: Selected DDR genes showing strong inverse correlation between promoter methylation and mRNA expression.** Only CpG probe sets (DDR genes of the reduced candidate gene list (see Additional file 3)) with a methylation frequency of >5% in tumor entities were included to ensure suitable sample number for statistical evaluation.
