# Supplementary material for: Promoter methylation of DNA damage repair (DDR) genes in human tumor entities: RBBP8/CtIP is almost exclusively methylated in bladder cancer
Source: Clin Epigenetics. 2018 Feb 6;10:15. doi: 10.1186/s13148-018-0447-6 (PMC5802064; doi:10.1186/s13148-018-0447-6)
Supplement: Supplementary file 5 — This table illustrates the clinicopathological parameters of 405 bladder cancer specimens of the TCGA network analyzed in this study. (DOC 48 kb) [file 13148_2018_447_MOESM5_ESM.doc]

| **Table S1: Clinico-pathological parameters of 405 bladder cancer specimens (TCGA) analyzed in this study** | | | |
| --- | --- | --- | --- |
|
|  | **Categorisation** | **n** | **% analyzable** |
|  |  |  |  |
| ***Parameter:*** |  |  |  |
| Gender |  |  |  |
|  | male | 259 | 73.4 |
|  | female | 94 | 26.6 |
|  | unknown | 52 | - |
| Histological tumor grade^c^ |  |  |  |
|  | low grade | 20 | 5.7 |
|  | high grade | 330 | 94.3 |
|  | unknown | 55 | - |
| Tumor stage^c^ | |  |  |
|  | pT1 | 2 | 0.5 |
|  | pT2 | 107 | 26.4 |
|  | pT3 | 168 | 51.7 |
|  | pT4 | 48 | 14.8 |
|  | unknown | 80 | - |
| Lymph node status |  |  |  |
|  | NX | 26 | - |
|  | N0 | 212 | 65.4 |
|  | N1 | 41 | 12.7 |
|  | N2 | 63 | 19.4 |
|  | N3 | 8 | 2.5 |
|  | unknown | 55 | - |
| Metastasis status |  |  |  |
|  | MX | 172 | - |
|  | M0 | 173 | 96.1 |
|  | M1 | 7 | 3.9 |
|  | unknown | 53 | - |
| ^a^Only patients with primary, bladder cancer without any neoadjuvant therapy were included. | | | |
|
