# Supplementary material for: Promoter methylation of DNA damage repair (DDR) genes in human tumor entities: RBBP8/CtIP is almost exclusively methylated in bladder cancer
Source: Clin Epigenetics. 2018 Feb 6;10:15. doi: 10.1186/s13148-018-0447-6 (PMC5802064; doi:10.1186/s13148-018-0447-6)
Supplement: Supplementary file 6 — This table summarizes the results from the multivariate Cox regression analysis including all factors influencing overall survival. (DOC 34 kb) [file 13148_2018_447_MOESM6_ESM.doc]

| **Table S2:** | | | | | | | |
| --- | --- | --- | --- | --- | --- | --- | --- |
| **Multivariate Cox regression analysis including all factors influencing OS** | | | | | | | |
| **Variable** | | |  | **HR** | **P-value** | **95%CI** | |
|  | | |  |  |  | **lower** | **upper** |
| RBBP8 methylation^a^ | | |  | 0.650 | 0.249 | 0.312 | 1.354 |
| Tumor stage | |  |  | 2.647 | **0.014** | 1.218 | 5.752 |
| pN status | | |  | 1.983 | **0.023** | 1.101 | 4.833 |
| pM status |  | |  | 1,410 | 0.584 | 0.412 | 2,809 |
|  | | | | | | | |
|
