# Supplementary material for: Promoter methylation of DNA damage repair (DDR) genes in human tumor entities: RBBP8/CtIP is almost exclusively methylated in bladder cancer
Source: Clin Epigenetics. 2018 Feb 6;10:15. doi: 10.1186/s13148-018-0447-6 (PMC5802064; doi:10.1186/s13148-018-0447-6)
Supplement: Supplementary file 7 — This table lists the RBBP8 methylation in human cell lines analyzed by MSP. (DOC 52 kb) [file 13148_2018_447_MOESM7_ESM.doc]

| **Table S3: RBBP8 methylation in human cell lines** | | |
| --- | --- | --- |
| **tissue of origin** | **cell line** | **RBBP8 methylation** |
|
| **Bladder** | UROtsa | U |
|  | RT4 | **M** |
|  | RT112 | **M** |
|  | EJ28 | U |
|  | J82 | U |
| **Kidney** | SKRC1 | U |
|  | SKRC10 | U |
|  | SKRC52 | U |
|  | SKRC59 | U |
| **Prostate** | LNCaP | U |
|  | RWPE-1 | U |
|  | PC3 | U |
|  | DU145 | U |
| **Lung** | H157 | U |
|  | H2170 | U |
|  | A549 | U |
| **Colon** | CaCo2 | U |
|  | CaCo205 | U |
|  | CaCo320 | U |
|  | SW480 | U |
|  | RKO | U |
|  | HT29 | U |
|  | HCT116 | U |
| **Breast** | HCC1937 | U |
|  | MDA-MB231 | U |
|  | ZR75-1 | U |
|  | MCF7 | U |
|  | T47D | U |
|  | SKBR3 | U |
|  | BT474 | U |
|  | MDA-MB468 | U |
|  | MCF10A | U |
|  | MCF12A | U |
|  | UACC-3199 | U |
|  | MDA-MB436 | U |
|  | BT20 | U |
