# Supplementary material for: Promoter methylation of DNA damage repair (DDR) genes in human tumor entities: RBBP8/CtIP is almost exclusively methylated in bladder cancer
Source: Clin Epigenetics. 2018 Feb 6;10:15. doi: 10.1186/s13148-018-0447-6 (PMC5802064; doi:10.1186/s13148-018-0447-6)
Supplement: Supplementary file 8 — This table shows the results of bivariate correlation statistics (Fisher exact test) between clinicopathological parameters and RBBP8 methylation. (DOC 49 kb) [file 13148_2018_447_MOESM8_ESM.doc]

| **Table S4:** | | | | |  |  |
| --- | --- | --- | --- | --- | --- | --- |
| **Clinico-pathological parameters in relation to RBBP8 methylation** | | | | | | |
|
|  | | **RBBP8 methylation^b^** | | | |  |
|  | | ***n****^a^* | **low** | **high** | **P-value**^c^ | **Spearman r** |
| ***Parameter:*** | | | | |  |  |
| Age at diagnosis | |  |  |  |  |  |
|  | <70 years | 13 | 8 | 5 | 0.613 | 0.113 |
|  | ≥70 years | 8 | 4 | 4 |
| Gender | | | | |  |  |
|  | male | 18 | 10 | 8 | 0.725 | 0.079 |
|  | female | 3 | 2 | 1 |
| Tumor subtype | | | | |  |  |
|  | non-invasive papillary | 8 | 7 | 1 | **0.031** | 0.481 |
|  | invasive | 13 | 5 | 8 |
| Histological tumor grade^d^ | |  |  |  |  |  |
|  | low grade | 7 | 7 | 0 | **0.006** | 0.612 |
|  | high grade | 14 | 5 | 9 |
| Tumor stage^d^ | |  |  |  |  |  |
|  | pT1-pT2 | 6 | 2 | 4 | 0.735 | -0.098 |
|  | pT3-pT4 | 7 | 3 | 4 |
|  |  |  |  |  |  |  |
| ^a^Only patients with primary, bladder cancer were included; ^b^score (IRS) according to Remmele and Stegner [74]; ^c^Fisher’s exact test; ^d^According to WHO 2004 classification; Significant P-values are marked in bold face. | | | | | | |
|
|
