# Supplementary material for: Promoter methylation of DNA damage repair (DDR) genes in human tumor entities: RBBP8/CtIP is almost exclusively methylated in bladder cancer
Source: Clin Epigenetics. 2018 Feb 6;10:15. doi: 10.1186/s13148-018-0447-6 (PMC5802064; doi:10.1186/s13148-018-0447-6)
Supplement: Supplementary file 9 — A table is shown characterizing 42 bladder cancer specimens analyzed in this study by IHC. (DOC 46 kb) [file 13148_2018_447_MOESM9_ESM.doc]

| **Table S5: Clinico-pathological parameters of 42 bladder cancer specimens analyzed in this study by immunohistochemistry** | | | |
| --- | --- | --- | --- |
|  |  |  |  |
|  | **Categorization** | **n^a^ analyzable** | **%** |
| ***Parameter:*** |  |  |  |
| Age at diagnosis: | median: 70 years |  |  |
|  | (range 26-94) |  |  |
|  | <70 years | 23 | 54.8 |
|  | ≥70 years | 19 | 45.2 |
| Gender |  |  |  |
|  | male | 33 | 78.6 |
|  | female | 9 | 21.4 |
| Tumor subtype |  |  |  |
|  | papillary non-invasive | 17 | 40.5 |
|  | invasive | 25 | 59.5 |
| Histological tumor grade^b^ | |  |  |
|  | G1 | 9 | 21.4 |
|  | G2 | 5 | 11.9 |
|  | G3 | 28 | 66.7 |
| Histological tumor grade^c^ | |  |  |
|  | low grade | 12 | 28.6 |
|  | high grade | 30 | 71.4 |
| Tumor stage^c^ |  |  |  |
|  | pTx | 1 | 2.4 |
|  | pTa | 16 | 38.1 |
|  | pT1 | 1 | 2.4 |
|  | pT2 | 12 | 28.6 |
|  | pT3 | 7 | 16.7 |
|  | pT4 | 5 | 11.9 |
| ^a^Only patients with primary bladder cancer were included; ^b^According to WHO 1973 classification; ^c^According to WHO 2004 classification. | | | |
