# Supplementary material for: Promoter methylation of DNA damage repair (DDR) genes in human tumor entities: RBBP8/CtIP is almost exclusively methylated in bladder cancer
Source: Clin Epigenetics. 2018 Feb 6;10:15. doi: 10.1186/s13148-018-0447-6 (PMC5802064; doi:10.1186/s13148-018-0447-6)
Supplement: Supplementary file 10 — This table summarizes the clinicopathological parameters of urine samples analyzed in this study by MSP. (DOC 48 kb) [file 13148_2018_447_MOESM10_ESM.doc]

| **Table S6: Clinico-pathological parameters of urine samples analyzed in this study by MSP** | | | |
| --- | --- | --- | --- |
|
|  | **Categorisation** | **n** | **% analyzable** |
|  |  |  |  |
| **Controls:** |  | 27 |  |
| Age (median 67) | |  |  |
|  | ≤67 years | 14 | 51.9 |
|  | >67 years | 13 | 48.1 |
| Gender |  |  |  |
|  | male | 22 | 81.5 |
|  | female | 5 | 18.5 |
| Diagnosis | |  |  |
|  | disease-free | 10 | 100 |
|  |  |  |  |
| **BLCA associated^a^** | | 22 |  |
| Age (median 72) | |  |  |
|  | ≤73.5 years | 11 | 50.0 |
|  | >73.5 years | 11 | 50.0 |
| Gender |  |  |  |
|  | male | 21 | 95.5 |
|  | female | 1 | 4.5 |
| Histological tumor grade^b^ | |  |  |
|  | low grade | 6 | 27.3 |
|  | high grade | 16 | 72.7 |
| Tumor stage^b^ | |  |  |
|  | pTa | 5 | 22.7 |
|  | pT1 | 7 | 31.8 |
|  | pT2 | 4 | 18.3 |
|  | pT3 | 5 | 22.7 |
|  | pT4 | 1 | 4.5 |
| ^a^Only urine samples derived from preoperative patients with primary, bladder cancer were included; ^b^According to WHO 2004 classification. | | | |
|
|
