# Supplementary material for: Promoter methylation of DNA damage repair (DDR) genes in human tumor entities: RBBP8/CtIP is almost exclusively methylated in bladder cancer
Source: Clin Epigenetics. 2018 Feb 6;10:15. doi: 10.1186/s13148-018-0447-6 (PMC5802064; doi:10.1186/s13148-018-0447-6)
Supplement: Supplementary file 13 — Illustrating the definition of probe sets near a TSS using three different genes (RBBP8, MGMT, and LIG4). (DOCX 1053 kb) [file 13148_2018_447_MOESM13_ESM.docx]

**
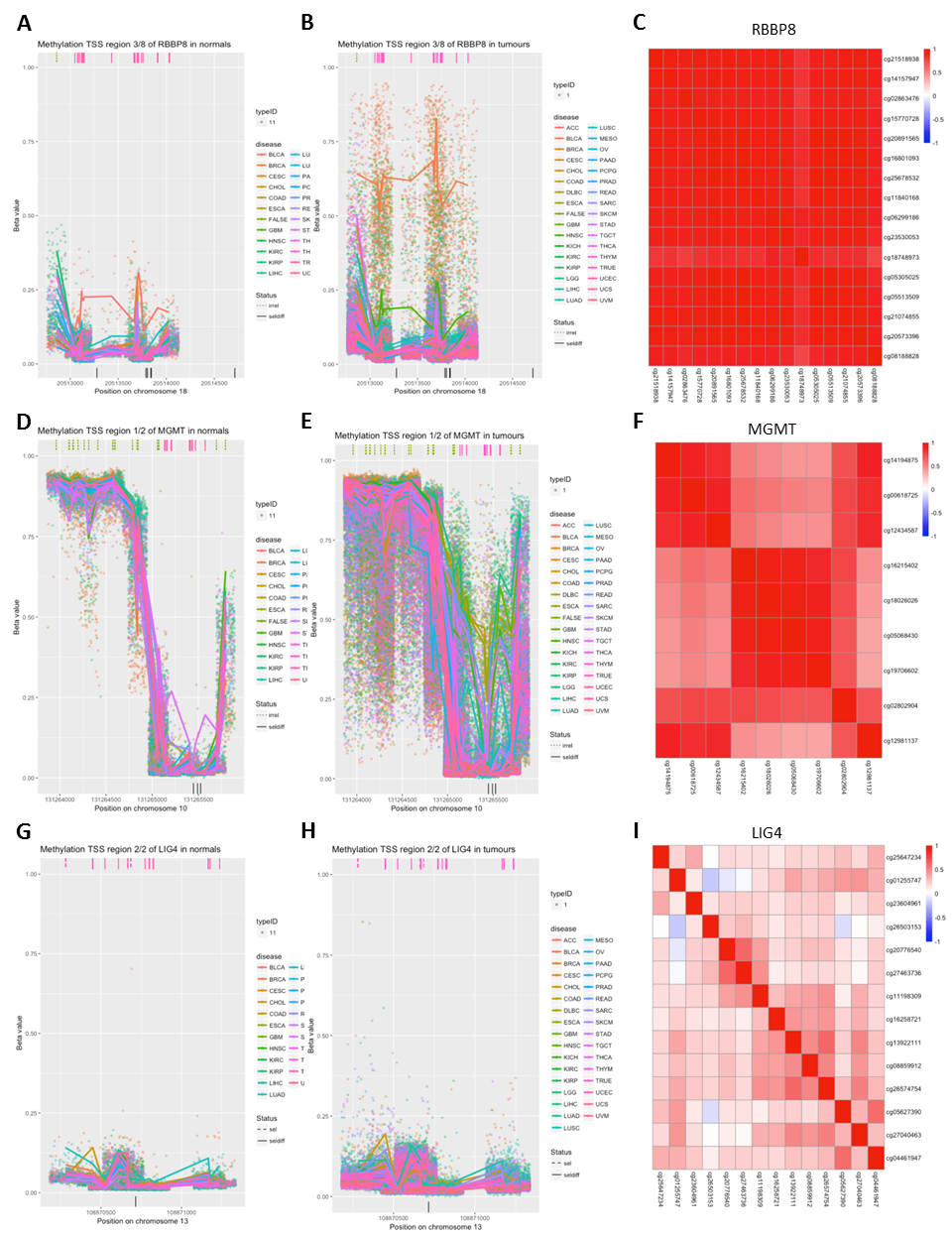
**

**Figure S5:** Illustration of probe set definition near a TSS based on the genes *RBBP8* **(A-C)**, *MGMT* **(D-F)**, and *LIG4* **(G-I)**. **(A,D,G)** Visualization of the methylation status in the genome region of RBBP8, MGMT, and LIG4 close to the TSS as found in normal tissues of 24 different organ types. For abbreviations see Table 1. **(B,E,H)** Entity-specific pattern of methylation for *RBBP8*, *MGMT* and *LIG4* in tumor tissues is illustrated. For abbreviations see Table 1. **(C,F,I)** Correlation coefficients between probes are shown for all three genes, respectively.
