# Supplementary material for: Promoter methylation of DNA damage repair (DDR) genes in human tumor entities: RBBP8/CtIP is almost exclusively methylated in bladder cancer
Source: Clin Epigenetics. 2018 Feb 6;10:15. doi: 10.1186/s13148-018-0447-6 (PMC5802064; doi:10.1186/s13148-018-0447-6)
Supplement: Supplementary file 15 — This table shows the sequences of all primers and conditions used in this study for MSP analysis. (DOC 33 kb) [file 13148_2018_447_MOESM15_ESM.doc]

| **Table S7 – Oligonucleotide**  **primers used in this study for methylation-specific PCR** | | |
| --- | --- | --- |
| **Gene** | **Sequence (5' → 3')** | **T_A_ (ºC)** |
| *RBBP8-U* | Forward: 5'-TTGTT AGATT TTTTG GGTAG TTTTT GGTAG TTTTG-3' | 60 |
|  | Reverse: 5'-CATAC CCTAA CTAAA AAACA AACTC TTCAA TACA-3' |
| *RBBP8-M* | Forward: 5'-CGTTA GATTT TTCGG GTAGT TTTCG GTAGT TTC-3' | 60 |
|  | Reverse: 5'-CGTTA GATTT TTCGG GTAGT TTTCG GTAGT TTC-3' |
| **MSP cycle conditions:** The PCR was initiated as "Hot Start" PCR at 94°C and held at 80°C before the addition of 1.25 units *Taq* DNA polymerase (Promega, Madison, WI) 95°C for 5 min, 35 (tissue) - 38 (urine) cycles of 95°C for 20 s, 60°C for 15 s, 72°C for 20 s and a final extension at 72°C for 10 min. | | |
|
|
|
