# Supplementary material for: Promoter methylation of DNA damage repair (DDR) genes in human tumor entities: RBBP8/CtIP is almost exclusively methylated in bladder cancer
Source: Clin Epigenetics. 2018 Feb 6;10:15. doi: 10.1186/s13148-018-0447-6 (PMC5802064; doi:10.1186/s13148-018-0447-6)
Supplement: Supplementary file 16 — This table lists the primer sequences for bisulfite-pyrosequencing of the RBBP8 promoter region. (DOC 32 kb) [file 13148_2018_447_MOESM16_ESM.doc]

| **Table S8:** | |  |
| --- | --- | --- |
| **Primer sequences for pyrosequencing** | | |
|  |  | |
| **Primer** | **Sequence (5' → 3')** | |
|
| ***RBBP8*  PCR For** | 5’-GGTTTGAGTTTTTTTTAGYGTAATTTAGAAATGTTGTGG-3’ | |
| ***RBBP8*  PCR Rev** | 5’-Bio-ACTTACRTCACRCCCCTTCCTCCAACT-3’ | |
|  |  | |
| ***Seq RBBP8*** | 5’-YGTAATTTAGAAATGTTGTGG-3’ | |
