# Supplementary material for: Promoter methylation of DNA damage repair (DDR) genes in human tumor entities: RBBP8/CtIP is almost exclusively methylated in bladder cancer
Source: Clin Epigenetics. 2018 Feb 6;10:15. doi: 10.1186/s13148-018-0447-6 (PMC5802064; doi:10.1186/s13148-018-0447-6)
Supplement: Supplementary file 17 — Sequences of all primers and conditions for qPCR analysis are summarized in a table. (DOC 32 kb) [file 13148_2018_447_MOESM17_ESM.doc]

| **Table S9 – Oligonucleotide primers used in this study for quantitative RT-PCR** | | | |
| --- | --- | --- | --- |
|
| **Gene** | **Sequence (5' → 3')** | **T_A_ (ºC)** | **Cycles** |
| *GAPDH* | Forward:  5’-GAAGGTGAAGGTCGGAGTCA-3’ | 60 | 40 |
|  | Reverse: 5’-TGGACTCCACGACGTACTCA-3’ |
| *RBBP8* | Forward: 5'-CCATC AACCT CTTCT ACGGC A-3' | 60 | 40 |
|  | Reverse: 5'-GCTTT TCACC AAGAG CAGAG G-3' |
| **Real-time PCR reaction volumes of 20 µl consisted of the following components:** | | | |
| 5 µM forward primer, 5 µM reverse primer, 10 µl SYBR GRN Supermix and 1 µl of cDNA as PCR template. Cycle conditions: 95°C for 3 min, 40 cycles of 95°C for 30 s, 60°C for 20 s, 72°C for 30 s. | | | |
|
|
